# PEGylated nicotinamide mononucleotide targeted ferroptosis in acute kidney injury by suppressing FAR1 palmitoylation in an ACAA1-dependent manner

**DOI:** 10.7150/ijbs.132234

**Published:** 2026-10-02

**Authors:** Yang Yue, Changdong Zhou, Qingyu Li, Xiaochang Zhang, Hongjun Lin, Ming Niu, Aihong Zhang, Shuiqin Gong, Lijing Yang, Yuke Cui, Hong Chen, Xiaodan Cheng, Jiangbo Li, Qi He, Chenhui Wang, Jian Li, Wen Yang, Jian Bai, Jingqi Shi, Yanze Li, Xing Qin, Xiangmei Chen, Liang Guo, Shengqi Wang

**Affiliations:** 1 Academy of Military Medical Sciences, Beijing 100850, China.; 2 Department of Nephrology, First Medical Center of Chinese PLA General Hospital, Beijing 100853, China.; 3 National Key Laboratoty of Kidney Diseases, National Clinical Research Center of Kidney Diseases, Beijing 100853, China.; 4 Pingyuan Laboratory, Henan 453000, China.; 5 Department of Hematology, the Fifth Medical Center of Chinese PLA General Hospital, Beijing 100071, China.; 6 Department of Nephrology, Chongqing Key Laboratory of Prevention and Treatment of Kidney Disease, Chongqing Clinical Research Center of Kidney and Urology Diseases, Xinqiao Hospital, Army Medical University (Third Military Medical University), Chongqing 400037, China.; 7 Medical Service Training Center, Chinese PLA General Hospital, Beijing 100853, China.; 8 CapitalBio Technology Corporation, Beijing 101111, China.

**Keywords:** acute kidney injury, NMN, ferroptosis, ACAA1, FAR1 palmitoylation

## Abstract

Acute kidney injury (AKI) remains a major health concern globally, with ferroptosis being the most critical pathological change in proximal tubule cells (PTCs). However, no specific pharmacotherapies have been developed yet. Here, by combining databases (*in vitro* LINCS and *in vivo* MTDS) covering drug-altered gene profiles with ferroptosis-associated FerrDb, nicotinamide mononucleotide (NMN) was identified to target renal ferroptosis. Furthermore, PEGylation increased the preferential uptake of NMN in mice renal cortex, enhancing its alleviating effect on AKI. Through MALDI-MS-based spatially resolved metabolomics and SEAM analyses, NMN-PEG was found to augment peroxisomal function in PTCs via ACAA1, leading to increased β-oxidation of LCFAs and VLCFAs to mitigate ferroptosis and inflammation. Notably, ACAA1 also suppressed palmitoylation of FAR1 Cys111 to mediate NMN-PEG-improved lipid metabolism. Our study is the first to reveal the renal ferroptosis-targeting role of NMN-PEG and the underlying molecular mechanism, shedding new light on the development of novel AKI therapeutics.

## Introduction

Acute kidney injury (AKI) is a frequent clinical challenge characterized by a sudden loss of excretory kidney function within 48 hours and is associated with substantial morbidity and mortality 1. More than 13 million individuals are affected by AKI annually, leading to 1.7 million deaths 2. Ischemia, inflammation (sepsis) and nephrotoxic substances are considered as the major causes of AKI 3, 4. However, no effective pharmacologic strategy for AKI has been well established, resulting in increased hospitalization costs and a prolonged length of stay. Thus, the identification of potential therapeutic targets and related drugs for AKI is urgently needed.

Renal tubular epithelia are particularly vulnerable to AKI through various forms of cell death 5. Ferroptosis, a novel type of regulatory cell death characterized by free iron overload and lipid peroxidation (LPO) 6, 7, is the most critical pathological change in proximal tubule cells (PTCs) upon AKI 8-10. Multiple laboratories, including ours, have been dedicated to exploring potential therapeutic targets and drugs by combining wet/dry approaches. The Library of Integrated Network-based Cellular Signatures (LINCS) 11 and our recently published Mouse Tissue-specific Drug Signatures (MTDS, http://drugtarget.cloudna.cn/) database, covering gene expression profile changes in response to different drugs *in vitro* and *in vivo*, respectively, provide valuable data for revealing the relationships between drugs and potential target genes. Additionally, ferroptosis-associated genes and substances were identified by FerrDb (zhounan.org), the first database that aims to reveal ferroptosis regulators and the associations between ferroptosis and disease. Making full use of these data would be beneficial for an efficient exploration of potential ferroptosis-associated genes and related drugs in multiple diseases.

Nicotinamide mononucleotide (NMN), a main precursor of nicotinamide adenine dinucleotide (NAD^+^), has been reported to efficiently inhibit ferroptosis in skin injury, aged mice, and heart failure 12-14. Mechanistically, NMN prevents ferroptosis by restoring intracellular NAD⁺ levels, which in turn recruits glutathione (GSH) to activate Glutathione peroxidase 4 (GPX4) and suppresses lipid peroxidation in UV-induced skin injury and cadmium-induced acute kidney injury 12, 15. Moreover, NMN inhibits CD38-induced ROS increase and DHFR degradation, leading to reduced ferroptosis in macrophages in aged mice 13. NMN administration has also been shown to ameliorate ferroptosis by improving lysosomal function and preventing iron accumulation within lysosomes in chronic heart failure 14. Of note, NMN has been also revealed as a potential therapeutic agent for AKI 15, 16. NMN has been demonstrated to reduce mitochondrial GPX4 acetylation and renal cell ferroptosis in a SIRT3-dependent pattern to mitigate Cadmium-induced AKI 15. NMN also alleviates age-related AKI through manipulating SIRT1 activity 16.

However, owing to the lack of kidney-targeted drug delivery strategies, high doses of NMN are used to achieve therapeutic effects, which usually induce side effects in peripheral tissues 17. Thus, engineered nanoparticles targeting proximal tubule cells (PTCs) are being developed for AKI treatment 18. PEGylation (the process of attaching a polyethylene glycol (PEG) polymer) to small molecules not only improves the water solubility of the drug but also promotes kidney-targeted transportation 2, 19-21. However, no data have been reported on the PEGylation of NMN or its potential for preferential renal uptake in AKI treatment.

Ferroptosis is caused by missed control of membrane lipid peroxidation, which occurs in a scenario of altered lipid homeostasis 22. Acetyl-coenzyme acyltransferase (ACAA1) is a peroxisomal acyltransferase involved in the β-oxidation of fatty acids to cleave 3-ketoacyl-CoA to acetyl-CoA and acyl-CoA, driving the extension and degradation of fatty acids 23. Moreover, it acts downstream in the peroxisome proliferator-activated receptor (PPAR) pathway to catalyze the synthesis of esterified cholesterol from long-chain fatty acids (LCFAs), very-long-chain fatty acids (VLCFAs) and free cholesterol 24. As ACAA1 is the key enzyme in the last step of β-oxidation 25, the loss of ACAA1 enzymatic activity decreases the rate of β-oxidation of LCFAs and VLCFAs, which induces polyunsaturated FA (PUFA) accumulation in lipid droplets to initiate ferroptosis 6. However, it still remains unknown whether ACAA1 is involved in ferroptosis of AKI and whether NMN modulates ferroptosis via ACAA1.

In this study, by matrix-assisted laser desorption/ionization-mass spectrometry (MALDI-MS)-based spatially resolved metabolomics and secondary ion mass spectrometry (SIMS)-based quantitative spatial single nuclear metabolomics (SEAM) analyses, NMN-PEG was found to successfully target lipid metabolism in cortical PTCs to inhibit ferroptosis. NMN-PEG improved β-oxidation of fatty acids in peroxisomes to reduce lipid accumulation and peroxidation, resulting in antiferroptotic and anti-inflammatory effects in AKI. Notably, ACAA1 was revealed to promote the degradation of fatty acyl-CoA reductase 1 (FAR1) by suppressing palmitoylation at Cys111, contributing to NMN-PEG-improved lipid metabolism in PTCs. Our study is the first to elucidate the antiferroptotic effect of NMN-PEG on PTC during AKI, offering considerable potential for the development of anti-AKI therapeutic strategy.

## Methods

### Animal studies

Following approval from the Animal Research Committee of AMMS, all animal experiments were performed in compliance with the NIH Guidelines on the Care and Use of Animals (IACUC-DWZX-2025-AP029), using C57BL/6J mice (6-8 weeks of age) obtained from Sipeifu Biotechnology (Beijing, China). The SLC12A8 knockout mice were purchased from Gempharmatech (Jiangsu, China). Mice were maintained under specific pathogen- free (SPF) conditions. To minimize confounding effects of sex, only adult male mice were used in this study. All animal experiments were performed with concealed allocation and blinding of outcome assessment.

### Cells lines

The human immortalized proximal tubule epithelial HK- 2 cells were obtained from the American Type Culture Collection and cultured at 37 ℃ in a 5% CO_2_ atmosphere. Upon reaching 80 % confluence, they were passaged for subsequent experiments. The cells were maintained in DMEM/F12 (PM150312, Procell) supplemented with 10% fetal bovine serum (F102-01, Vazyme) and 1 % penicillin/streptomycin (15140122, Gibco).

### Synthesis of PEGylated NMN

NMN (HY-F0004) was purchased from MedChemExpress. Briefly, 100 mg NMN was dissolved in 5 mL pyridine with succinic anhydride (1.05 equivalents) and DMAP (0.5 equivalents), and incubated at room temperature overnight. Then the reaction liquid was concentrated under reduced pressure, precipitated with acetone, filtered after washing with acetone to obtain NMN-COOH.

Then 70 mg NMN-COOH was dissolved in 2 mL water with NH_2_-PEG2000 (1.05 equivalents), PyBOP (1.3 equivalents) and DIPEA (5.0 equivalents). Then the solution was mixed with 5 mL dimethylformamide (DMF), and reacted at room temperature for 0.5 h. The reaction liquid was concentrated under reduced pressure and precipitated with ice ether. Finally, the PEGylated NMN was filtered and collected after washing with ice ether.

### Characterization of PEGylated NMN

PEGylated NMN was dissolved in anhydrous ethanol. A 20 μL aliquot of the sample was deposited onto a carbon-coated copper grid and left for 3-5 min, after which excess liquid was removed by blotting with filter paper. The grid was then stained with 2% phosphotungstic acid for 1-2 min. The copper grids were examined using a transmission electron microscope (TEM) (HT7800/HT7700, Hitachi, Japan). Dynamic light scattering (DLS) measurements were performed on a Zetasizer Pro (Malvern Panalytical, UK) at room temperature.

### Establishment of AKI models *in vivo*

IRI-induced AKI model: Mice were anesthetized via intraperitoneal injection of 1% pentobarbital, and bilateral renal pedicles were occluded using non-traumatic vascular clamps for 30 min to induce ischemia. Throughout the ischemic phase, body temperature was sustained at 37℃ using a heating pad. Following reperfusion (clamp release), the surgical incisions were sewn up, and animals were returned to their home cages with free access to food and water for 48 h prior to subsequent analyses 26.

LPS-induced AKI model: An intraperitoneal injection of lipopolysaccharide (LPS; 10 mg/kg) was administered to establish the sepsis-associated AKI model 27.

### Treatment of PEGylated NMN *in vivo*

In the IRI-AKI experiments, mice were randomly divided into 4 groups: Sham mice group; IRI mice group; IRI mice intravenously injected with NMN (500 mg/kg) at 2, 24 h group and IRI mice intravenously injected with NMN-PEG (20 mg/kg) group at 2, 24 h. All mice were sacrificed at 48 hours after IRI. The serums were obtained for monitoring the levels of BUN and SCr.

For LPS-induced AKI, NMN (500 mg/kg) or NMN-PEG (20 mg/kg) was administered intravenously 30 min after LPS challenge, whereas control animals received an equivalent volume of normal saline. At 24 h post-LPS injection, mice were euthanized. Serum was harvested for BUN and SCr measurement, and renal tissues were excised for histopathological evaluation.

### Safety evaluation of PEGylated NMN* in vitro*

HK-2 cells were seeded into 96-well plates at 1 × 10^4^ cells per well and treated with NMN-PEG at graded concentrations (1, 5, 10, 20, 50, 100, and 200 µg/mL) for 24 h. Following treatment, the culture supernatant was replaced with fresh medium, and 10 μL of CCK-8 solution (A311, Vazyme) was added to each well. After 1 h of incubation at 37℃, absorbance at 450 nm was recorded using a microplate reader (BioTek, USA).

### Safety evaluation of PEGylated NMN *in vivo*

To assess systemic safety, healthy C57BL/6J mice received daily intravenous NMN-PEG (20 mg/kg) for two consecutive days. Cohorts were euthanized on day 7 or day 30 post-injection. Whole blood was drawn for complete blood counts, serum was separated for biochemical assessment of hepatic and renal function, and tissues (heart, liver, spleen, lung, kidney, and intestine) were harvested for hematoxylin and eosin (H&E) histological examination.

### Distribution of PEGylated NMN *in vivo*

Both healthy and IRI-AKI mice received intravenous NMN-PEG (20 mg/kg) at 2 h and 24 h. At 48 h, animals were euthanized and major organs—heart, liver, spleen, lung, kidney, and intestine—were excised for ex vivo fluorescence imaging using a VILBER FUSION FX7 system (VILBER, France) to evaluate the biodistribution of NMN-PEG. To determine renal retention kinetics, healthy and IRI-AKI mice were injected with NMN-PEG (20 mg/kg, i.v.), and kidneys were collected at serial time points (3, 6, 12, 24, 48, and 72 h) for fluorescence imaging 28.

### Hemolytic assay

Whole blood of healthy mice was collected with anticoagulant tubes, centrifuged at 3000×*g* for 15 min, blood cells were collected and resuspended in normal saline to prepare a 4% red blood cell suspension. Different concentrations of 1 mL (25-100 µg/mL) NMN-PEG solution were mixed with 100 µL blood cells respectively. And 1mL ultra-pure water as positive control and 1mL PBS as negative control. The samples were incubated at 37℃ for 2 h and centrifuged at 3000×*g* for 15 min. Then the supernatant was used to determine OD value at 542 nm with a microplate reader (BioTek, USA). Hemolysis ratio = (OD of PEGylated NMN - OD of negative control) / (OD of positive control - OD of negative control) × 100% 29.

### HR induced AKI *in vitro* models

For HR model, HK-2 cells were cultured in an anoxic environment with 1% O_2_ for 24 h with HBSS replacement medium. Then, cells were transferred to normal incubators for 2 h, and were treated with NMN-PEG for 24 h in normal culture environment 30, 31.

### GSH, GSSG and MDA content assay

GSH and GSSG levels in HK-2 and mice kidneys tissue were measured with total glutathione/oxidized glutathione test kit (G4304, Servicebio) following the manufacturers' instructions. MDA content was detected with Malondialdehyde (MDA) test kit (G4300, Servicebio).

### RNA-seq

RNA extraction utilized RNeasy Mini Kit (Qiagen), following the manufacturer's direction. RNA integrity assessment was performed using Agilent 2100. A MGI platform was used for library sequencing, and 150 bp paired-end reads were generated. Differential expression analysis utilized the DESeq2 R package. *p* < 0.05, FC > 1.2 indicated significant differential expression.

### Cell transfection

The ACAA1 small interfering RNA (siRNA) and the FAR1 C111S mutant plasmid were synthesized by GenePharma. siRNAs were transfected into HK-2 cells using Lipofectamine RNAiMAX Reagent (13778100, Thermo Fisher), following the manufacturer's direction, and plasmid transfection into HK-2 cells was performed using X-tremeGENE™ HP DNA transfection reagent (41106502, Sigmaaldrich) following the manufacturer's instructions.

### ROS level detection

Culture medium was aspirated, and cells were washed and then incubated in PBS supplemented with 10 µM DCFH-DA probe (G1706, Servicebio). Fluorescent signals were captured at an excitation wavelength of 488 nm and an emission wavelength of 525 nm.

### Lipid droplet and lipid ROS detection

The number of lipid droplets in HK-2 was detected using BODIPY493/503 methyl bromide (GC68792, GLPBIO). The lipid ROS was detected by BODIPY-581/591 C11 (HY-D1301, MedChemExpress). Briefly, 10 μM BODIPY working solution was incubated with HK-2 cells for 30 min to stain the lipid droplets or lipid ROS. Then lipid droplets were observed by LSM 880 confocal microscopy (Carl Zeiss) and lipid ROS was detected using BD Aria III (BD Biosciences).

### Oil red O staining

HK-2 cells were fixed in 4% paraformaldehyde for 30 min and washed twice with PBS. Cells were permeabilized with a permeabilization buffer for 15 min, incubated with Oil Red O working solution for 30 min, and counterstained with hematoxylin for 30 seconds. After rinsing with deionized water, slides were mounted in glycerol-water (1:1, v/v) and photographed using an EVOS M5000 imaging system (Thermo Fisher).

Cryosections of kidney tissue were stained with freshly prepared and filtered Oil Red O solution for 10 min, briefly differentiated in 60% isopropanol, and counterstained with hematoxylin. After bluing, sections were mounted with glycerol gelatin and imaged by EVOS M5000 fluorescence imaging system (Thermo Fisher).

### Histopathology

Tissue specimens from heart, liver, spleen, lung, kidney, and intestine were fixed in 4% paraformaldehyde (G1101, Servicebio), paraffin-embedded, and cut into 4-μm sections. Sections were stained with a Hematoxylin-Eosin (H&E) staining kit (G1076, Servicebio), dehydrated, and examined under a Nikon light microscope for pathological evaluation. Renal tubular damage was semiquantitatively graded on a 0-5 scale based on the following criteria: tubular dilation, tubular epithelial necrosis, cast formation, and brush border loss. Scores of 0, 1, 2, 3, 4, and 5 corresponded to approximately 0%, <10%, 10-25%, 26-50%, 51-75%, and >75% of the affected tubular area, respectively 32.

### Immunofluorescence analysis

For tissue sections, after dewaxing and hydration, the kidney tissue sections were heated for antigen repair and then incubated with sodium citrate solution (G1202, Servicebio) and 3% H_2_O_2_ for membrane permeability and peroxidase removal. Nonspecific binding sites were blocked with 3% bovine serum albumin (BSA) (GC305010, Servicebio) and incubated with primary antibodies against AQP1 (P010883, Epizyme) at 4 ºC overnight. Then, the sections were incubated with Cy5-conjugated goat anti-rabbit IgG secondary antibody (GB27303, Servicebio) for 1 h at room temperature, and cell nuclei were stained with DAPI (G1012, Servicebio). For multiplex immunofluorescence staining, sections were incubated with primary antibody for 2 h, followed by detection using HRP-conjugated secondary antibody and TSA-fluorophores (G1257, Servicebio). The primary and secondary antibodies were eliminated by heating the slides. AQP1, AQP2 (P103145, Epizyme), SLC12A3 (ab95302, Abcam), SLC12A1 (18970-1-AP, Proteintech) and SLC12A8 (20308, BiCell Scientific) were sequentially detected. Vectashield containing DAPI nuclear counterstain was used to mount the sections. The slices were observed under an optical microscope (Nikon).

For cellular immunofluorescence, HK-2 cells growing on glass coverslips were immobilized with paraformaldehyde, and subsequently inoculated at room temperature utilizing 3% BSA for 30 min for blocking the non-specific binding sites. The slides of cells were incubated overnight with primary antibody anti-PMP70 (EPR5614, Abcam) at 4℃. Then cells were incubated with FITC-conjugated goat anti-rabbit IgG secondary antibody (GB22303, Servicebio) for 1h at room temperature. Cells were then stained with DAPI at ambient temperature for 10 min and were imaged using a LSM 980 inverse laser scanning microscope (Carl Zeiss).

### Total RNA extraction and RT-qPCR

RNA was extracted from mouse kidney tissue and HK-2 cell pellets using the Tissue Total RNA Isolation Kit V2 (RC112-01, Vazyme). Complementary DNA (cDNA) was synthesized via reverse transcription using HiScript III All-in-one RT SuperMix (R423-01, Vazyme). Quantitative real-time PCR was performed with Taq Pro Universal SYBR qPCR Master Mix (Q712-022, Vazyme). β-Actin served as the internal reference gene, and relative expression levels were computed using the 2^-∆∆Ct^ method. Primer sequences are provided in Tables S1 and S2.

### Western blot

Total protein of HK-2 cells was extracted with RIPA lysis buffer (P0013B, Beyotime) with protease and phosphatase inhibitor cocktails (P1046, Beyotime), and prepared for electrophoresis sample. After gel electrophoresis with Super-PAGE^TM^ prefabricated adhesive, the target protein on the gel was transferred to PVDF membrane, respectively. The PVDF membrane was sealed with 5% skim milk powder for 2 h at room temperature, and incubated with rabbit anti-ACAA1 (12319-2-AP, Proteintech), PMP70 (EPR5614, Abcam), FAR1 (PA5-53585, Thermo Fisher), ubiquitin polyclonal antibody (10201-2-AP, Proteintech) and mouse anti-GAPDH (60004-1-Ig, Proteintech), Flag (66008-4-Ig, Proteintech) antibodies at 4℃ overnight, respectively. After TBST rinsing, membranes were incubated with HRP Polyclonal Goat Anti-Rabbit IgG (ZB-2301, ZSGB-BIO) or HRP Polyclonal Goat Anti-Mouse IgG (ZB-2305, ZSGB-BIO) at room temperature for 1 h. ECL luminescent solution and VILBER FUSION FX7 gel imaging system (Vilber) were used for obtaining images.

### Transmission electron microscope observation

HK-2 cells and kidney tissue specimens were immersed in electron microscopy fixation solution at 4℃, followed by post-fixation in 1% osmium tetroxide (in 0.1 M phosphate buffer, pH 7.4) for 2 h at ambient temperature in the dark. Samples were then dehydrated through a graded ethanol series, cleared in propylene oxide, and embedded in Spurr resin. Ultrathin sections (90 nm) were contrast-stained with 2% uranyl acetate in saturated ethanol and lead citrate, and examined on an HT7700 transmission electron microscope (Hitachi).

### MAlDI platform-based spatial metabolism

The instrument, times TOF flex MALDI was used to carry out MSI experiments. The data were acquired at a maximum rate of 10,000 Hz in full scan with positive and negative ionization modes from *m/z* 100 to 1050. The size of laser spot was set to 50 µm in the single mode. Metabolite annotation was performed by matching accurate mass values (within 10 ppm tolerance) against the HMDB (version 5.0) and METLIN (https://metlin.scripps.edu) databases. Metabolites were assigned at a mass accuracy of less than 10 ppm. Significantly changed compounds were selected with* P* < 0.05.

### SIMS platform-based SEAM

Fresh kidney tissues were embedded in OTC for SEAM analysis. TOF-SIMS 5 coupled with a Bi liquid metal ion gun (30 keV Bi_3_^+^LMIG with an HSR mode) was used for creating spectra and images of kidney 33. The secondary ion images were obtained with Bi_3_^+^LMIG over a 400 × 400 µm^2^ area with 256 × 256 pixels. CH_2_^-^, O^-^, OH^-^ and PO2^-^ were used for negative spectra. To mitigate charge accumulation, a low-energy electron flood gun was employed, while depth profiling was performed using a 10-keV Ar2500+ commercial gas cluster ion gun as the sputtering source. The final 2D image was generated by overlaying 182-184 layers of depth profiling scans. Peak identification was conducted according to the recommended SurfaceLab parameters (mass range: 50-500; minimum counts: 10,000), with each peak representing a spatially resolved ion image. HPLC-grade metabolite standards were introduced into TOF-SIMS and analyzed under conditions identical to those of the HMR mode. Furthermore, adenine (*m/z* 134.04) served as the nuclear marker for segmenting regions of interest (ROI).

Correlation between metabolite expression and distance from the damaged RTECs was calculated through the following steps using R package EBImage (version 4.44.0): (1) “cell cut with key metabolite” image (Document S1) was converted into green channel. (2) The image was smoothed using Perreaults' modern constant-time filtering algorithm 34. (3) “fillHull” was used to detect the edge of damaged RTECs. (4) The closest euclidean distance from each spot to the edge of the damaged RTECs was calculated. (5) The Pearson correlation between the expression level of each spot and the distance to the damaged RTECs was performed to identified the damaged-related *m/z*.

Seurat was utilized to integrate and analyze the SEAM expression profile. Cluster was identified by the function “FindClusters” under the resolution of 2. Each cluster was annotated based on the spatial location.

### High-throughput targeted metabolic assay

Renal cortical tissues dissected from mouse kidneys were snap-frozen and stored. Samples were thawed gradually at 4 ºC, and aliquots were homogenized in a pre-chilled methanol-acetonitrile-water mixture (2:2:1, v/v). Following centrifugation, the supernatant was collected and lyophilized in a vacuum concentrator at 4 ºC. Metabolite profiling was conducted on an Agilent 1290 Infinity UHPLC system interfaced with an AB Sciex TripleTOF 6600 mass spectrometer to acquire MS1 and MS2 spectra. Statistical significance between groups was evaluated by unpaired Student's t-test, with *P* < 0.05 as the threshold.

### Enzyme-linked immunosorbent assay (ELISA)

The levels of palmitic acid (MM-926334O2, Mmbio) and palmitoyl-CoA (MM-61547H2, Mmbio) in HK-2 cells were determined using assay kits in accordance with the manufacturer's instructions.

### Quantitative palmitoyl-proteomics

Mouse kidney tissues were pulverized under liquid nitrogen and homogenized in lysis buffer containing 4% SDS, 100 mM Tris-HCl (pH 8.0), 5 mM EDTA, and protease inhibitor cocktail (Thermo Fisher). The lysates were sonicated on ice for 2 min followed by centrifugation at 4 ºC to collect supernatants. Following protein quantification, equal protein amounts from each sample were subjected to SDS-PAGE electrophoresis. For palmitoylation analysis, qualified protein samples underwent free thiol blocking followed by methanol-chloroform-water precipitation. The precipitated proteins were resuspended in lysis buffer and equally divided into hydroxylamine-treated (HA^+^) and control (HA^-^) groups. The HA^+^ group was incubated with 1 M hydroxylamine to specifically reduce palmitoylated cysteine residues, while the HA^-^ group received no hydroxylamine treatment. Both groups were then labeled with 10 mM Cys-Tag (CPT) reagent at 37 ºC for 2 h in the dark to tag newly liberated thiols, followed by another methanol-chloroform-water precipitation. The precipitated proteins were dissolved in 50 mM NH_4_HCO_3_ and digested with trypsin (600×*g* for 1 min, then 16-18 h at 37 ºC). Digested peptides were acidified with 100% TFA, centrifuged, and the supernatant was collected and dried by vacuum centrifugation. CPT-labeled peptides were enriched using the High-Select Fe-NTA Phosphopeptide Enrichment Kit (Thermo Scientific) according to the manufacturer's protocol, followed by C18 cartridge desalting and lyophilization.

For LC-MS/MS analysis, peptides were separated on a Vanquish Neo UHPLC system using a PepMap Neo C18 trap column (5 µm, 300 µm × 5 mm) and μPAC Neo High Throughput analytical column with a gradient of 0.1% formic acid in water (A) versus 0.1% formic acid in 80% acetonitrile (B): 4-8% B over 0.1 min, 8-10% B over 1.1 min, 10-28% B over 10 min, 28-45% B over 2.3 min, and 99% B for 1.5 min. Mass spectrometry was performed on an Orbitrap Astral instrument in data-independent acquisition (DIA) mode with positive ionization (2.2 kV), MS1 scans at 240,000 resolution (380-980 m/z), and MS2 scans at 80,000 resolution using HCD fragmentation with 25% normalized collision energy, 2 Th isolation windows, and 0.6 s cycle time. The spectra of full MS scan and DIA scan were recorded in profile and centroid type respectively.

### scRNA-seq

A previously obtained scRNA-seq dataset was downloaded from GEO database (GSE197266). Then, the expression matrices were integrated using “anchor-based” integration workflow in the R package Seurat (version 4.3.0). The “FindMarker” function of the Seurat package was used to identified the differentially expressed genes.

Gene Ontology (GO) and KEGG pathway enrichment analysis were conducted by the function “enrichGO” and “enrichKEGG”, which were provided using R package ClusterProfiler (version 4.6.2). The Benjamini-Hochberg (BH) method was used for the multiple test adjustments.

### Acyl-biotin exchange assay

The acyl-biotin exchange (ABE) assay was conducted following established protocols with slight modifications 35, 36. Briefly, cells were lysed on ice for 30 min using RIPA lysis buffer (P0013B, Beyotime) supplemented with protease and phosphatase inhibitor cocktails (P1046, Beyotime). The resulting whole-cell lysate (WCL) supernatant, collected by centrifugation at 12,000 × g for 10 min at 4 ºC, was subjected to protein concentration determination via the bicinchoninic acid (BCA) assay. Subsequently, protein samples were mixed with 2 mM N-ethylmaleimide (NEM; HY-D0843, MedChemExpress) and antibodies targeting FAR1 (PA5-53585, Thermo Fisher) or FLAG (66008-4-Ig, Proteintech), and incubated at 4℃ for 3 h. This was followed by an overnight incubation with Protein A/G PLUS-Agarose beads (sc-2003, Santa Cruz) at 4 ºC. Afterward, the beads were rinsed with lysis buffer and divided into two aliquots: one was treated with hydroxylamine (HAM; 467804, Sigma), while the other was treated with PBS containing protease/phosphatase inhibitors; both treatments were carried out at room temperature for 1 h. Following a wash step with PBS containing 0.05% Tween-20 and inhibitors, the samples were labeled with Biotin-BMCC (C100222, BBI) for 1 h at room temperature. Finally, proteins were eluted in 1 × SDS loading buffer by heating at 100 ºC for 10 min, and the eluates were analyzed by immunoblotting with HRP-streptavidin (A0303, Beyotime) and anti-FAR1 or anti-FLAG antibodies.

### Immunoprecipitation

The immunoprecipitation assay was performed using the Protein A/G immunoprecipitation kit (YJ201, Epizyme). Briefly, cells were lysated in IP lysis buffer and phosphatase inhibitor cocktails (P1046, Beyotime). The mixture was incubated on ice for 20 min and then centrifuged at 12,000×*g* for 10 min at 4 °C. The resulting supernatants were incubated with agarose-conjugated anti FAR1 (PA5-53585, Thermo Fisher) or FLAG (66008-4-Ig, Proteintech) antibody overnight at 4 ºC. The immunoprecipitated beads were washed, eluted with loading buffer and boiled at 100 ºC for 10 min. After cooling, these samples were subjected to western blot analysis.

### Immunopurification-mass spectrometry

HK-2 cells stably expressing FLAG-tagged FAR1 were harvested and lysed in IP buffer. The cleared lysate was incubated with 10 μg of anti-FLAG antibody overnight at 4 ºC. Protein A/G agarose beads were pre-washed with lysis buffer, added to the antibody-lysate mixture, and incubated overnight at 4 ºC. Beads were pelleted by centrifugation (2,500 rpm, 3 min, 4 ºC), the supernatant was discarded, and the beads were washed three times with 1 mL of lysis buffer. Bound proteins were eluted, resolved by SDS-PAGE, and visualized by silver staining. Gel bands of interest were excised and subjected to LC-MS/MS analysis by genecreate.cn (Wuhan, China).

### Quantification and statistical analysis

Data are presented as the mean ± standard deviation (SD). Differences among groups were assessed using one-way analysis of variance (ANOVA). Statistical analyses were performed with GraphPad Prism (Version 9.0), and a *P*-value of less than 0.05 was considered statistically significant.

## Results

### NMN was predicted to be an effective anti-ferroptosis agent in the kidney by multiple database-based drug screening

To explore potential drugs that target ferroptosis in the kidney, we performed a large-scale screening using both *in vitro* (LINCS) and *in vivo* (MTDS) drug-induced gene expression profile databases based on three clusters of ferroptosis-related genes extracted from FerrDb (ferroptosis suppressors, ferroptosis drivers and unclassified ferroptosis-related genes) (Figure 1A). A total of 1592 and 14 significant compounds were identified from the MTDS (Figure 1B) and LINCS (Figure 1C), respectively, with 4 overlapping compounds (NMN, aspirin, metformin, and andrographolide) (Figure 1D). Consistent with published literatures revealing the potential protective roles of NMN against AKI, among the top 10 significantly enriched treatments of the kidney by MTDS, 3 involved the administration of different doses of NMN (Figure 1B), indicating the critical role of NMN in affecting ferroptosis-associated genes. Furthermore, the differentially expressed genes (DEGs) in mice treated with NMN (24 h, 500 mg/kg) (Figure 1E&F) were further assayed by Gene Ontology (GO) and Kyoto Encyclopedia of Genes and Genomes (KEGG) enrichment analyses. NMN treatment affected mainly the processes of ferroptosis, lipid metabolism, and ROS production, including fatty acid oxidation, PPAR signaling pathway, and peroxisome pathway (Figure 1G&H). And thus, NMN was selected for subsequent study.

On the basis of a multiple database screening strategy, NMN was identified as a promising antiferroptosis drug in the kidney that significantly affects the expression profile of ferroptosis-related genes.

### PEGylated NMN preferentially accumulated in the PTCs of AKI mouse models

Low-molecular-weight PEGylation improves water solubility and the preferential renal transport of small molecules 37. Therefore, we proposed the development of PEGylated NMN to combat the ferroptosis of PTCs. PEGylated NMN nanoparticles (NMN-PEG) were obtained by mixing carboxylated NMN with NH_2_-PEG2000 to allow their self-assembly (Figure 2A&B, Figure S2A). The physicochemical properties of PEGylated NMN nanoparticles were verified by their stability and ultrasmall size (200 nm) (Figure 2C&D). We further assessed the toxicity and biocompatibility of NMN-PEG both *in vitro* (Figure 2E&F) and *in vivo* (Figure S1). No hemolytic toxicity was detected when the concentration of NMN-PEG in the blood was below 50 µg/mL (Figure 2E). Moreover, no evident pathological changes detected in the major organs (heart, liver, lung, spleen, lung, kidney and intestine) of mice intravenously injected with NMN-PEG (20 mg/kg) for 7 and 30 days (Figure S1A). Routine blood tests revealed no significant differences in liver function or kidney function between the control and NMN-PEG-treated mice (Figure S1B).

To facilitate visualization of the distribution of NMN-PEG *in vivo*, PEGylated NMN was labeled with FITC. Compared to NMN without modification, PEGylated NMN significantly enhanced it renal targeting capacity, especially in IRI-induced AKI mouse models. NMN-PEG was taken mainly into the kidney, liver, and intestine, with the highest uptake detected in the kidney derived from an ischemia/reperfusion injury (IRI)-induced AKI mouse model (Figure 2G&Figure S2B). The fluorescence intensity of the kidney peaked at 12 hours of treatment and then was maintained until 24 h (Figure 2H, Figure S2C). More importantly, compared with tubular cells from other nephron segments, NMN-PEG preferentially accumulated within the renal cortex of mouse models and co-expressed with aquaporin 1 (AQP1)-positive proximal tubule cells in IRI-induced AKI mouse models (Figure 2I, Figure S2D).

Our data demonstrated that, via PEGylation, NMN-PEG, without obvious toxicity, successfully targeted PTCs in the AKI mouse models.

### PEGylated NMN alleviated IRI- and LPS-induced ferroptosis of PTCs both *in vitro* and* in vivo*

To evaluate the potential therapeutic effect of PEGylated NMN *in vitro*, the human proximal tubule cell line HK-2 was subjected to hypoxia-reoxygenation (HR) injury, an *in vitro* pathological change closely associated with IRI, or LPS stimulation, both of which are major causes of AKI. Afterward, the cells were treated with a gradient concentration of NMN-PEG for 24 hours (Figure S3A). The viability of HK-2 cells in response to HR was increased by NMN-PEG in a dose-dependent manner (Figure S3B). In the mouse models of IRI- and LPS-induced AKI (Figure 3A, Figure S4A), the increase in kidney size was dramatically diminished by NMN-PEG treatment, with reduced proximal tubule damage and lower serum creatinine (SCr) and blood urea nitrogen (BUN) levels (Figure 3B-D, Figure S4B-D).

Additionally, we measured kidney injury biomarkers and inflammatory cytokines and revealed that the IRI (HR)- or LPS-enhanced expression of kidney injury molecule 1 (KIM1), neutrophil gelatinase-associated lipocalin (NGAL), tumor necrosis factor alpha (TNF-ɑ), interleukin (IL)-6, and IL-1β were markedly decreased by NMN-PEG both *in vitro* (Figure S3C&D) and *in vivo* (Figure 3E&F; Figure S4E&F). Notably, via transmission electron microscopy (TEM) and Oil Red O staining, the typical characteristics of ferroptosis, manifested by mitochondrial shrinkage, mitochondria membrane rupture, cristae reduction, and lipid accumulation were observed in IRI/LPS-induced AKI mice and HR-treated HK-2 cells and were alleviated by NMN-PEG (Figure 3G&H; Figure S4G&H; Figure S3E). Consistent with these findings, IRI/LPS and HR significantly activated redox and lipid peroxidation, as evidenced by increased level of malondialdehyde (MDA) and a reduced ratio of glutathione (GSH)/glutathione disulfide (GSSG), which were reversed by NMN-PEG (Figure 3I-L; Figure S4I-L; Figure S3F&G).

These data revealed that NMN-PEG efficiently inhibited ferroptosis in IRI- and LPS-induced ferroptosis by targeting PTCs.

### PEGylated NMN upregulated ACAA1 to enhance peroxisome function

To decipher the potential molecular mechanisms underlying NMN-PEG-mediated alleviation of AKI, we performed RNA-seq analysis of control HK-2 cells and HR-treated HK-2 cells treated with or without PEGylated NMN (Figure 4A). Volcano plots of differentially expressed genes (DEGs) were shown in Figure 4B&D. KEGG analysis revealed that the peroxisome pathway was among the top 20 enriched pathways in both the control *vs.* HR and NMN-PEG-treated HR *vs.* HR groups (Figure 4C&E), indicating that PEGylated NMN improved HR-disturbed peroxisome function. Furthermore, the heatmap of peroxisome-associated DEGs based on the RNA-seq data revealed that the expression of peroxisomal genes was inhibited mainly by HR but significantly restored by NMN-PEG (Figure 4F).

To identify potential hub genes involved in AKI-induced peroxisome damage, we analyzed previously published scRNA-seq data from multiple AKI mouse models (Figure S5A). Different cell populations were clustered by marker genes (Figure S5B&C), among which PTCs, the most vulnerable cell type in AKI, were focused for further analysis. Consistent with the results of the RNA-seq analysis, GO and KEGG analyses revealed that the DEGs in all five mouse models of AKI were related to abnormal fatty acid metabolism and peroxisomes (Figure S5D). Moreover, acetyl-CoA acyltransferase 1A (*Acaa1a*), the gene homologous to *ACAA1* in mice, was significantly downregulated in PTCs in all the AKI models (Figure S5E). Moreover, ACAA1 was also the most upregulated gene in HR-stimulated HK-2 cells upon NMN-PEG treatment (Figure 4F). Further experiments revealed that HR stimulation reduced the expression of ACAA1 and 70-kDa peroxisomal membrane protein (PMP70), which could be reversed by NMN-PEG treatment (Figure 4G&H). However, the inhibition of ACAA1 in HK-2 cells by siRNA (siACAA1) neutralized the ability of NMN-PEG to restore HR-damaged peroxisomal function (Figure 4H&I). Peroxisomes play crucial roles in lipid metabolism by mediating β-oxidation of LCFAs and VLCFAs and synthesis of ether lipids such as plasmalogens, which directly influence lipid droplet formation and triglyceride accumulation 38-40. Upon ACAA1 knockdown, we found that neither lipid droplet accumulation nor triacylglycerol (TG) accumulation caused by HR could be decreased by NMN-PEG (Figure 4J&K). Similarly, NMN-PEG-mediated decreases in the levels of intracellular ROS and lipid peroxidase (labeled with BODIPY 581/591, a lipid peroxidation sensor) upon HR treatment were also increased by knockdown of ACAA1 (Figure 4L&M).

Our data suggested that HR induced significant damage to peroxisome function and fatty acid metabolism, which could be mitigated by NMN-PEG through increasing the expression of ACAA1. Moreover, ACAA1-regulated metabolites, especially LCFA/VLCFA, that played potential roles in AKI and targeted by NMN-PEG attracted our interest (Figure 4N).

### PEGylated NMN reduced LCFA/VLCFA accumulation and peroxidation in the PTCs of AKI mice

Subsequently, to identify critical metabolites, we applied MALDI‒MSI to detect the nontargeted spatial metabolome of the freshly dissected kidneys of IRI-induced AKI mice treated with or without NMN-PEG (Figure 5A). The two groups were clearly distinguished by partial least squares-discriminant analysis (PLS-DA) (Figure S7A&B). Approximately 2755 metabolites and 53 differentially expressed metabolites (*P* < 0.05; VIP > 1), including alcohols, amines, aldehydes, ketones, esters, amino acids, benzene, FAs, glycerolipids (GL), sphingolipids (GP), hormones, organic acids, and saccharolipids (SL), were revealed (Figure 5B). Since NMN-PEG preferentially accumulated in the cortex of mouse kidneys (Figure 2G&I), we focused on the metabolic profile of the renal cortex. PEGylated NMN modulated the metabolism of diverse lipids and acylcarnitines (including triacontanedioic acid, traumatic acid, and myristoylcarnitine) in the renal cortex of AKI mice, with a particularly significant downregulation of oxidized lipids (Figure 5C&D).

Furthermore, we performed targeted metabolome analysis of the renal cortex derived from AKI mice treated with or without NMN-PEG (Figure 5E, Figure S7C&D). Compared with the IRI-induced AKI model controls, the NMN-treated mice exhibited significant reductions in 19 LCFAs, 7 VLCFAs, and 5 carnitine derivatives (including palmitelaidic acid, palmitic acid, and palmitoylcarnitine) (Figure 5F&G).

In conclusion, combining the nontargeted spatial metabolome of kidney sections and the targeted metabolome of the isolated renal cortex, NMN-PEG efficiently reversed lipid metabolism disorders in the renal cortex induced by AKI, which was responsible for the antiferroptotic effect of NMN-PEG in the AKI mouse models.

### SEAM revealed *in situ* metabolic reprogramming of epithelial cells in the renal cortex in NMN-PEG-treated mice

Spatial single nuclear metabolomics (SEAM) analysis is a novel integrated platform for qualitative and quantitative analysis of the spatial single nuclear metabolomics of the tissue microenvironment *in situ* 33. As an advanced imaging mass spectrometry (IMS) method, time-of-flight-secondary ion mass spectrometry (TOF-SIMS) was used for mass spectrometry and the generation of ion images of tissue sections. Chemical information was extracted from a 400×400 µm^2^ area on kidney sections from the sham and AKI mice treated with or without NMN-PEG and output as multiplexed SIMS data with a spatial resolution of 256×256 pixels. In accordance with the analysis methods employed by Yuan et al., single-nucleus segmentation was achieved using SIMS-Cut, which segments regions of interest on the basis of corresponding metabolic markers. Additionally, single-nucleus representation was implemented via SIMS-ID, which constructed a bipartite graph of pixels and cells using a self-supervised learning algorithm to effectively characterize individual cells. A total of 182, 182, and 184 ions were detected in the sham, AKI, and NMN-PEG-treated AKI mice, respectively, with the corresponding nuclear counts being 606, 558, and 620, respectively, in each group (Figure 6A). The SIMS-Cut-detected nuclei in the field of view and the SIMS-ID representing each cell with a fixed vector length were fed into the SIMLR module 41 for metabolic distinct cell clustering.

To better resolve spatial cell annotation *in situ*, H&E staining, total ion images and SIMS-View were matched within the same field of view to obtain a spatial single-nuclear metabolic map, which revealed renal tubular epithelial cells (RTECs) and glomerular cells as the most strongly affected cell types in the cortex (Figure 6B-D). The percentage of each cell subpopulation in the different groups was quantified (Figure 6E). We further measured the gray distribution of the total ion images from each group to evaluate spatial cortical damage. NMN-PEG treatment decreased the density of the low-scale ion intensity peak (damaged area) but increased that of the high-intensity peak (undamaged area) compared with those in AKI mouse models (Figure 6F).

By combining the m/z feature analysis with the distance to the damaged area, m/z 81.01 was detected to spread from the undamaged area toward the damaged area. 3D surface visualization also indicated that m/z 81.01 was enriched within the damaged area (Figure 6G). To identify the exact metabolites contributing to IRI-induced tubular damage, standards of metabolites that were differentially expressed in the renal cortex of the NMN-PEG-treated AKI group versus the AKI group were analyzed using TOF-SIMS (Figure S6). The changes in the ion species, including the one at m/z 81.01, were consistent with those of the metabolite standards, especially palmitic acid, palmitelaidic acid, and palmitoylcarnitine (Figure 6H-I, Figure S6). Particularly, m/z 81.01 was identified as a characteristic peak of palmitoylcarnitine (Figure S6B).

And thus, the accumulation of palmitoylcarnitine (m/z 81.01) in kidney upon AKI indicated a disorder of mitochondrial fatty acid β-oxidation, along with peroxisomal FAO dysfunction induced by ACAA1-downregulation. NMN-PEG treatment reduced m/z 81.01 in the injured region to restore lipid metabolism function.

And thus, consistent with the results from MALDI-MSI and targeted metabolome analysis, SEAM further confirmed that PEGylated NMN reversed the accumulation of VLCFAs/LCFAs, especially palmitic acid-associated metabolites, in the PTCs.

### PEGylated NMN promoted FAR1 protein degradation through reducing S-palmitoylation at Cys111 in an ACAA1-dependent manner

To further investigate the renal-protective mechanisms of NMN-PEG, we performed a quantitative proteomics analysis of IRI-induced AKI models (Figure 7A). Volcano plot analysis revealed that Far1 was significantly increased by AKI while decreased by NMN-PEG treatment (Figure 7B). FAR1 has been demonstrated to catalyze the reduction of C16 or C18 saturated fatty acids into fatty alcohols, a critical step in the biosynthesis of alkyl ether lipids and plasmalogens 42. Particularly, very recently, FAR1 has been demonstrated to be involved in ferroptosis. Inactivation of FAR1 attenuates saturated fatty acid-dependent ferroptosis, and FAR1-mediated ferroptosis relies on peroxisome-driven ether phospholipid biosynthesis 43. Consistent with our previous transcriptomics findings, KEGG analysis of the differentially expressed proteins revealed that peroxisome pathway was among the top 10 significantly enriched pathways in both the sham *vs*. AKI and the NMN-PEG-treated AKI *vs*. untreated AKI group comparisons (Figure 7C&D). These findings endowed a potential nexus role of FAR1 in linking lipid remodeling to peroxisome-dependent cell death.

Intriguingly, by Liquid Chromatography-Tandem Mass Spectrometry (LC‒MS/MS) analysis, a novel palmitoylation site at Cys111 (C111) of Far1 was identified (Figure 7E). This aroused our interest as palmitic acid-associated metabolites were revealed to be significantly varied in opposite patterns under AKI and NMN-treated condition, respectively. Further investigation in HK-2 cells using acyl-biotin exchange (ABE) assay confirmed that HR induced FAR1 palmitoylation, which was suppressed by NMN-PEG (Figure 7F). As the disorders of palmitic acid metabolism in AKI was revealed to be mediated by ACAA1, we further analyzed the potential role of ACAA1 in FAR1 palmitoylation. As shown in Figure 7F, ACAA1 knockdown reversed the inhibitory effect of NMN-PEG on FAR1 palmitoylation (Figure 7F). Additionally, cycloheximide (CHX) treatment demonstrated that ACAA1 knockdown markedly attenuated FAR1 protein degradation in HK-2 cells (Figure 7G). However, this protective effect was diminished when Cys111 was mutated to serine (C111S) in FAR1 (Figure 7G). Consistently, C111S mutation markedly enhanced the ubiquitination of FAR1, indicating that S-palmitoylation restrained FAR1 degradation through inhibiting its ubiquitination (Figure 7H). Furthermore, ACAA1-dependent reduction of FAR1 by NMN-PEG in HK2 cells upon HR stimulation was also abolished by C111S mutation, while MG132, the proteasome inhibitor, blocked the inhibitory effect of NMN-PEG, which demonstrated that S-palmitoylation protects FAR1 against proteasomal degradation (Figure 7H&I).

To elucidate the upstream mechanism by which ACAA1 inhibits FAR1 palmitoylation, we further conducted immunoprecipitation-mass spectrometry (IP-MS) analysis to identify FAR1-interacting proteins. The results showed that the interaction between FAR1 and the core palmitoylation enzymes APT1 or ZDHHC14 was not significantly altered by HR treatment or NMN-PEG treatment in HK-2 cells (Figure S8A&B). Furthermore, the intracellular levels of the palmitoylation substrate were analyzed. We found that NMN-PEG treatment significantly reduced the levels of both palmitic acid (PA) and its activated form, palmitoyl-CoA (P-CoA), which were reversed by knockdown of ACAA1 (Figure S8C&D).

Collectively, these findings demonstrate that NMN-PEG upregulates ACAA1 expression, leading to a reduction in the substrate pool of P-CoA and then a suppression of HR-induced FAR1 palmitoylation at Cys111. This inhibition of palmitoylation subsequently enhances the proteasomal degradation of FAR1, contributing to the reduction of ferroptosis (Figure 7J).

### Cellular uptake of PEGylated NMNs was mediated by the *Slc12a8* transporter

Solute Carrier Family 12 Member (SLC12A8) has been well established as a *bona fide* NMN transporter, and genetic ablation or functional deficiency of *Slc12a8* significantly impairs cellular NMN uptake 44. To investigate whether NMN-PEG similarly depends on SLC12A8-mediated transport, we generated* Slc12a8*-knockout (KO) mice (Figure 8A). Compared to the wild-type mice, *Slc12a8*-KO mice significantly blocked the mitigating effect of NMN-PEG on the tubular damage of renal upon IRI treatment (Figure 8B). Moreover, NMN-PEG-reduced serum level of SCr and BUN, as well as the mRNA levels of AKI biomarkers (*Kim1* and *Ngal*) and inflammatory cytokines (*Tnf-α*, *Il-6*, and *Il-1ß*), were increased by *Slc12a8* knockout (Figure 8C-E).

NMN-PEG-alleviated mitochondrial ferroptosis and lipid accumulation in the renal cortex of IRI mice were reversed in *Slc12a8*-KO mice, indicating that the anti-ferroptosis role of NMN-PEG was mediated by SLC12A8 (Figure 8F&G). NMN-PEG also failed to reverse the increased level of MDA and the reduced ratio of GSH/GSSG in *Slc12a8*-KO mice (Figure 8H-K). Furthermore, in the IRI-induced AKI mouse model, SLC12A8 was primarily co-localized with AQP1of the proximal tubule cells, supporting that the uptake of NMN-PEG by proximal tubule cells was mainly mediated by SLC12A8 (Figure S8E).

In conclusion, our data demonstrated that, similar with unmodified NMN, the therapeutic role of PEGylated NMN in AKI also relied on SLC12A8-mediated cellular uptake.

## Discussion

AKI, characterized by an acute decrease in renal function, remains a major health concern globally 45. It is well documented that the protection of RTECs against ferroptosis is effective in reducing the mortality of AKI 46, 47. In this study, by employing a PEGylation strategy, NMN preferentially accumulated in the RTECs of AKI mouse models, leading to the alleviation of LPS- or IRI-induced AKI. Furthermore, NMN-PEG enhanced peroxisome function through ACAA1-dependent inhibition of FAR1 palmitoylation, leading to reduced ferroptosis upon AKI.

PEGylation is regarded as an efficient way to reduce kidney clearance of molecules by linking one or more PEG chains, providing a modification strategy for enhancing the assimilation of drugs in the treatment of renal diseases 48. Recently, the fundamental role of ferroptosis in AKI has become a concern 49, 50. In this study, NMN was identified as an overlapping anti-ferroptosis molecule in the kidney between MTDS and LINCS (Figure 1B). Furthermore, PEGylation of NMN obtained a preferential renal uptake in mouse models, which reduced the effective dose to 20 mg/kg and enhanced the anti-inflammatory/antiferroptotic effect of NMN in AKI (Figure 3F-L).

RNA-seq and scRNA-seq data revealed that ACAA1 functioned as a core factor in lipid peroxidation and the resulting ferroptosis in AKI. Previous studies revealed that ACAA1 is a critical member of the PPAR pathway and fatty acid metabolism and is responsible for VLCFA/LCFA β-oxidation 25. Dysfunction of ACAA1 decreased the rate of peroxisomal β-oxidation of palmitoyl-CoA 51. Our data showed that AKI-dysregulated LCFA and VLCFA metabolism was improved by NMN-PEG through upregulating ACAA1 to enhance peroxisome function (Figure 4).

To better understand the changes in lipid metabolism status, especially the spatial metabolomic changes upon NMN-PEG treatment in IRI-induced AKI, multiple metabolomics analyses were performed. MALDI-based spatially resolved metabolomics analysis confirmed that PEGylated NMN preferentially accumulated in the cortex of AKI mice and influenced the fatty acid metabolism, especially fatty acylcarnitine and oxidized lipids, leading to reduced lipid peroxidation (Figure 5B-D). Targeted metabolomic analysis of the renal cortex further confirmed the downregulation of LCFAs, VLCFAs, and carnitines by NMN-PEG in AKI (Figure 5F&G). More importantly, we utilized a novel SIMS-based SEAM to reveal the metabolic features of single cells and identified different RTEC metabolic subpopulations as well as their spatial organization 33. Metabolites involved in fatty acid oxidation (FAO), especially palmitic acid, palmitelaidic acid, and palmitoylcarnitine, were shown to be regulated by NMN-PEG via SEAM analysis, indicating the pro-FAO effect of PEGylated NMN in AKI. Notably, we also took advantage of this high-resolution single nuclear metabolic profiling to explore tubular damage-related metabolites and NMN-PEG-induced metabolic changes by calculating the intensity of ions of interest spatially (Figure 6). Though SEAM is a novel integrated platform for analyzing the spatial single nuclear metabolomics of the tissue microenvironment *in situ*, there is no established reference database for confidently annotating the metabolite profiles of specific tubular subtypes. And thus, data from SEAM should be analyzed together with other results. We found that the changes in the ion species were consistent with those of the metabolite standards, particularly palmitic acid, palmitelaidic acid and palmitoylcarnitine (Figure 6H-I, Figure S6). These data also supported the findings from targeted metabolomics analysis of isolated proximal tubule cells (Figure 5E-F).

Very recently, FAR1 has been reported to induce ferroptosis through the peroxisomal pathway of ether lipid biosynthesis 43. In this study, quantitative proteomics analysis revealed that FAR1 protein was upregulated in IRI-induced AKI but significantly downregulated after NMN-PEG treatment. Intriguingly, palmitoylation of FAR1 in PTCs to inhibit protein degradation by ubiquitination was identified, which was consistent to accumulation of palmitic acid-associated metabolites during AKI. Fatty acids are essential for S-acylation because they are substrates for acyl-CoA synthetase 52, and lipid metabolic disorder-induced palmitoylation is one of the most important type of S-acylation type 53. Moreover, ACAA1 knockdown reversed the inhibitory effect of NMN-PEG on FAR1 palmitoylation at Cys111, suggesting that the inhibition effect of NMN-PEG on FAR1 palmitoylation was dependent on ACAA1. Additionally, HR-induced S-palmitoylation at Cys111 impeded the ubiquitin‒dependent degradation of FAR1. Our data revealed, for the first time, that ACAA1-dependent palmitoylation of FAR1 functioned to bridge lipid metabolism with ferroptosis in AKI.

Conclusively, we identified NMN as a ferroptosis-targeted drug by multi-database analysis and modified NMN with PEG to improve its renal preference. NMN-PEG promoted the β-oxidation of fatty acids in peroxisomes to reduce lipid accumulation and peroxidation, leading to antiferroptotic and anti-inflammatory effects in both IRI- and LPS-induced AKI. Mechanistically, NMN-PEG, also transported by the *Slc12a8* protein, suppressed FAR1 palmitoylation in an ACAA1-dependent manner to improve FAR1 degradation, which reprogrammed lipid metabolism by enhancing the peroxisomal function and ultimately reduce ferroptosis in renal PTCs during AKI. Our study is the first to reveal the antiferroptotic effect of NMN-PEG on PTCs in AKI and the underlying molecular mechanism, shedding new light on the development of anti-AKI therapeutics.

## Supplementary Material

Supplementary figures and tables.

## Figures and Tables

**Figure 1:**
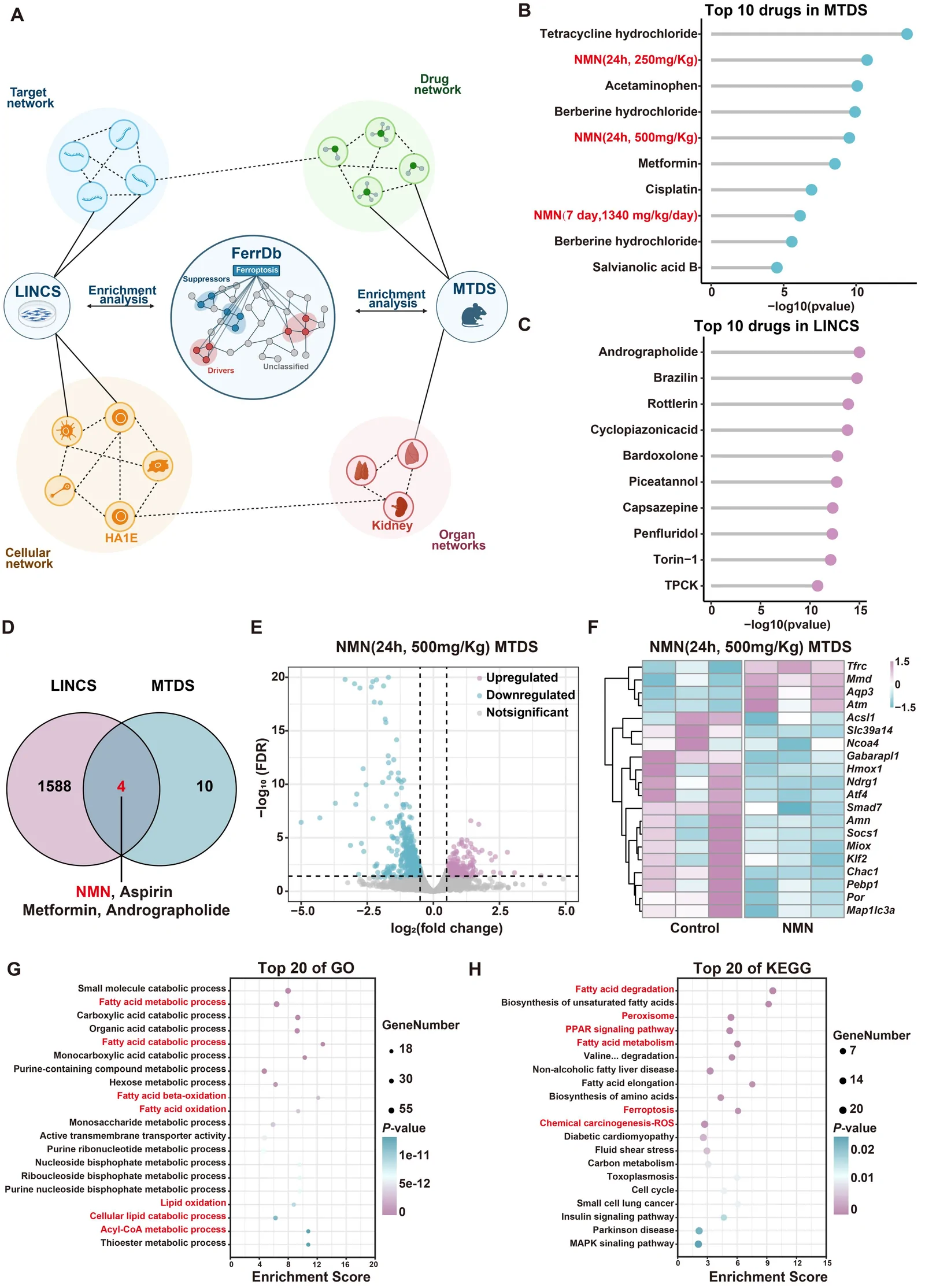
** NMN was identified by multidatabase screening to target ferroptosis in the kidney.** (A) Schematic of the drug screening strategy based on multiple databases. Renal ferroptosis-targeting drugs were predicted by combined analysis of databases that cover gene expression profile changes in response to different compounds *in vivo* (MTDS) and *in vitro* (LINCS) with the ferroptosis-associated gene and molecular database FerrDb. (B, C) The top ten enriched ferroptosis-targeting compounds in the kidney according to MTDS (B) and LINCS (C). The top enriched molecules in the MTDS or LINCS were identified on the basis of the three clusters of ferroptosis-related gene profiles derived from FerrDb (ferroptosis suppressors, ferroptosis drivers and unclassified ferroptosis-related genes). (D) Overlapping ferroptosis-targeting compounds between the LINCS and MTDS databases. Four reagents (NMN, aspirin, metformin, and andrographolide) were identified. (E-H) RNA-seq analysis of mice treated with or without NMN. The mice were treated with or without 500 mg/kg NMN for 24 hours before RNA-seq analysis. Volcano plot (E) and heatmap (F) analyses of the DEGs in NMN-treated mice *vs*. control mice are shown. GO term (G) and KEGG pathway (H) analyses were also performed. Lipid metabolism-associated pathways, including fatty acid metabolism, beta-oxidation, peroxisome, and the PPAR pathway, were enriched in NMN-PEG-treated mice.

**Figure 2:**
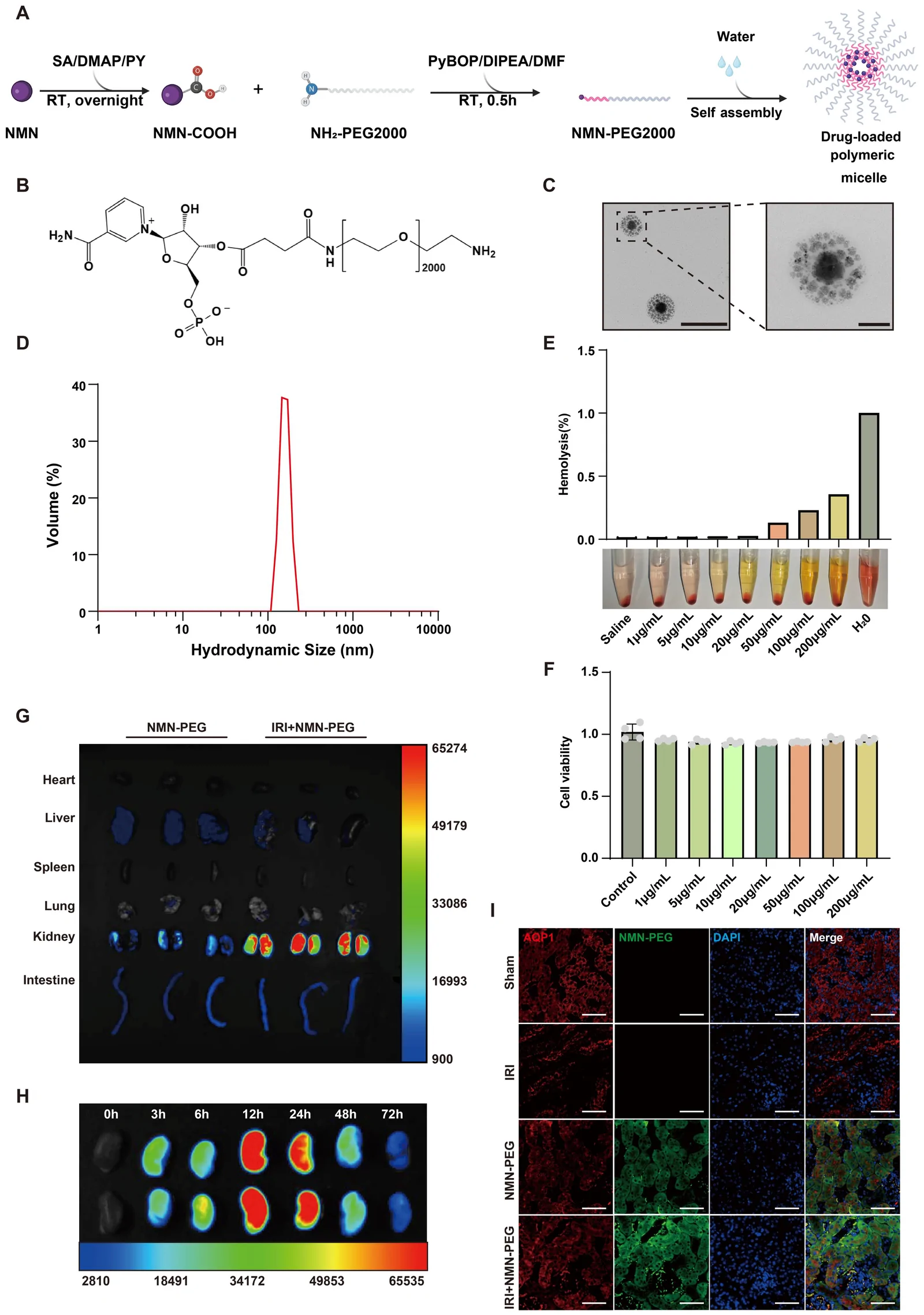
** PEGylation of NMN and its preferential uptake in the cortex of the kidney in AKI mouse models.** (A) Schematic of the NMN-based PEGylation strategy. Briefly, NMN, dissolved in pyridine, was mixed with succinic anhydride and 4-dimethylaminopyridine (DMAP) completely to make NMN-COOH, followed by reaction with NH2-PEG2000-FITC (or NH_2_-PEG2000), PyBOP, and diisopropyl ether (DIPE) in dimethylformamide (DMF) to obtain NMN-PEG2000-FITC or NMN-PEG2000 (NMN-PEG, A1). The NMN-PEG monomer self-assembled into polymeric micelles in water (A2). (B) The structure of NMN-PEG. (C, D) Biophysical characterization of PEGylated NMN. Representative transmission electron microscopy (TEM) images of PEGylated NMN (C, left scale bar: 200 nm; right scale bar: 20 nm). Dynamic light scattering (DLS) of the PEGylated NMN micelles was performed with a Zetasizer Pro (D). (E) Analysis of the hemolysis activity of NMN-PEG. The peripheral blood of C57BL/6 mice was centrifuged and resuspended in saline to obtain a 4% red blood cell suspension. One milliliter of PEGylated NMN at different concentrations was mixed with 100 μL of blood cell suspension and incubated at 37 °C for 2 h. The supernatant was subjected to OD450 analysis. (F) Effect of PEGylated NMN on the viability of HK-2 cells. HK-2 cells were seeded in a 96-well plate and exposed to different concentrations of NMN-PEG for 24 h, after which they were subjected to a CCK8 analysis (n = 6). (G-I) PEGylated NMN preferentially accumulated in the renal cortex of AKI mice. Representative fluorescence images of major organs (heart, liver, spleen, lung, kidney and intestine) after PEGylated NMN injection in control and AKI mouse models (G). Representative fluorescence images of the kidneys of mice at different time points (3, 6, 12, 24, 48, and 72 h) after the injection of NMN-PEG are shown (H). Representative images of PEGylated NMN (green) and aquaporin 1 (AQP1, red) in the kidneys of NMN-PEG-treated control and AKI mice are also displayed (I). Scale bar: 20 μm.

**Figure 3:**
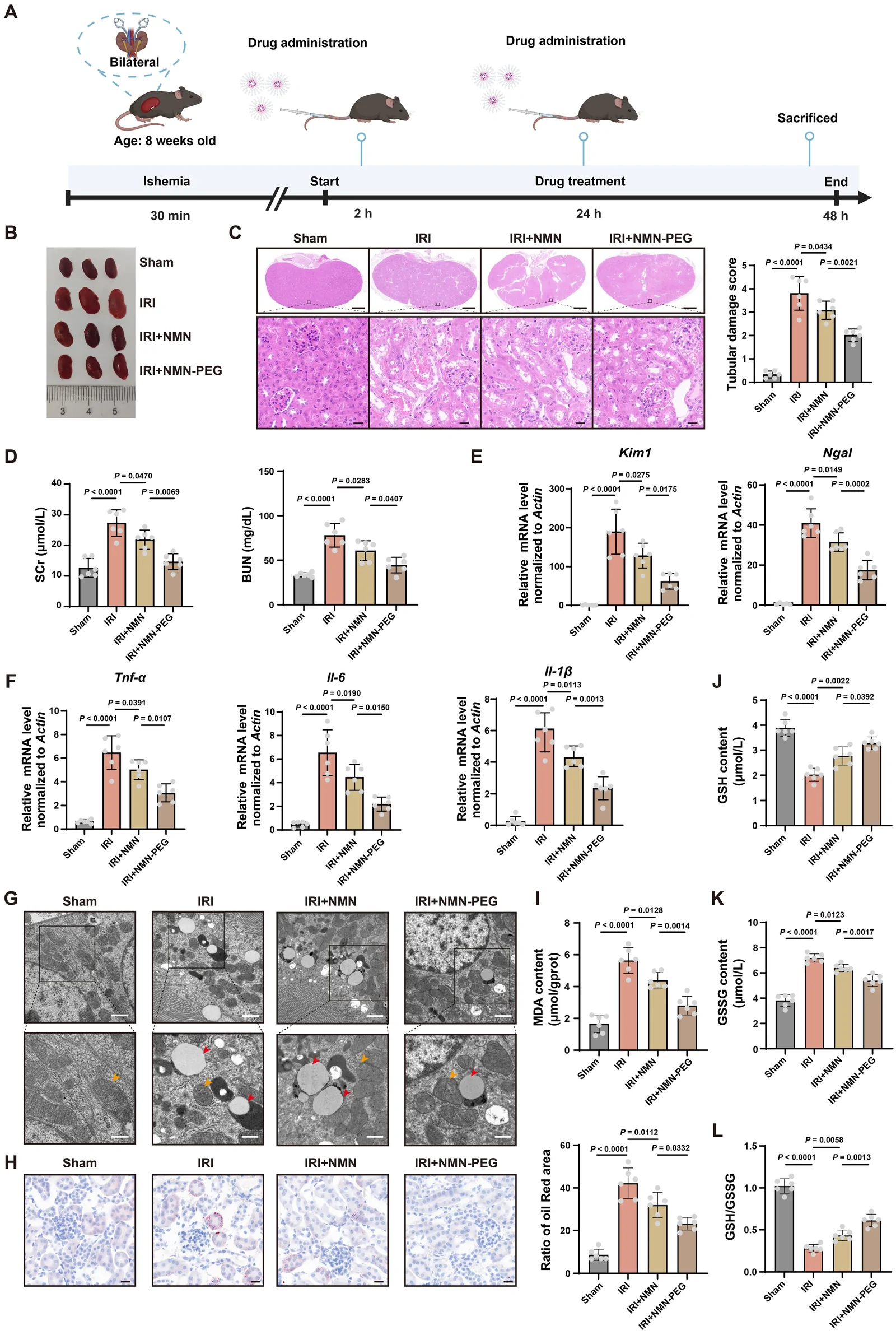
** NMN-PEG alleviated IRI-induced pathological changes and ferroptosis *in vivo.*** (A) Schematic diagram of the construction of the IRI-induced AKI mouse model and NMN administration. In brief, after surgical clamping of the bilateral renal pedicles for 30 min, the blood supply was restored in the mice to establish an IRI-induced AKI model. The mice were injected with NMN (500 mg/kg) or PEGylated NMN (20 mg/kg) at the indicated times after reperfusion and then sacrificed after 48 h. (B-F) PEGylated NMN reduced renal injury and inflammation in IRI-induced AKI mouse models. The kidneys of the sham and IRI mouse models treated with NMN or NMN-PEG at the indicated time points were collected after 48 hours. The macroscopic and H&E images of the kidney are shown in (B) and (C, n = 6; upper scale bar: 1000 μm; lower scale bar: 20 μm), respectively. The serum levels of blood urea nitrogen (BUN) and serum creatinine (SCr) in the mice in the different groups were assayed (D, n = 6). The mRNA levels of AKI biomarkers (*Kim1* and *Ngal*) (E) and inflammatory cytokines (*Tnf-α*,* Il-6*, and *Il-1β*) (F) in the kidneys derived from different groups of mice were analyzed (n = 6). (G-L) PEGylated NMN inhibited ferroptosis in mice with IRI-induced AKI. Representative TEM images of kidney mitochondria from different groups of mice (G, upper scale bar: 1.0 μm; lower scale bar: 0.5 μm). Mitochondria are indicated by yellow arrows. Lipid droplet is indicated by red arrows. Representative Oil Red O-staining images of kidney in different groups of mice (H, scale bar: 20 μm). The content of MDA in the kidney tissues of the mice was analyzed (I). Renal expression of GSH and GSSG and the ratio of GSH/GSSG in mice from different groups (J-L). (n = 6). The data are presented as the means ± SDs.

**Figure 4:**
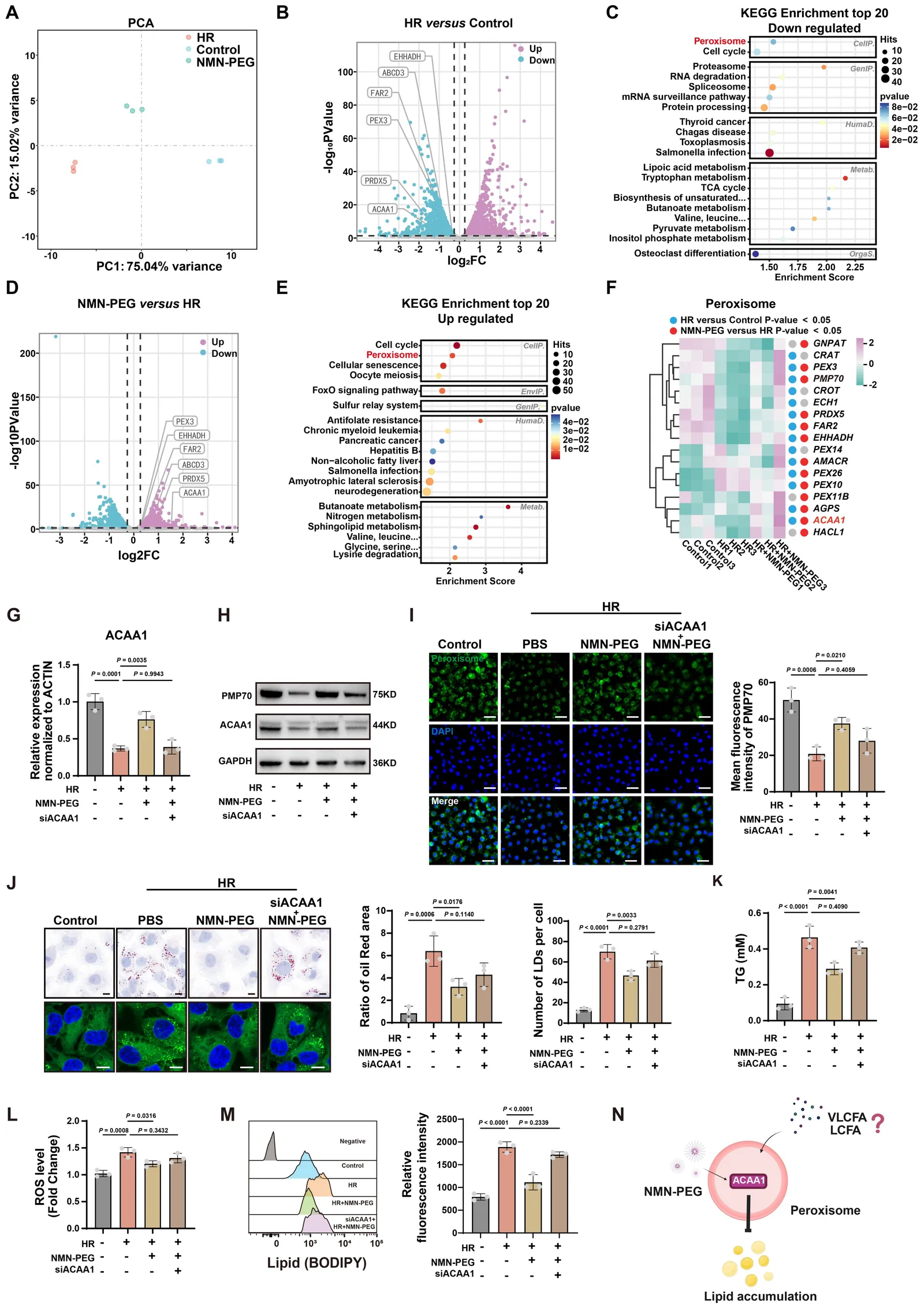
** PEGylated NMN regulated the function of peroxisomes through ACAA1.** (A) PCA of the RNA-seq data of control cells and HR-stimulated HK-2 cells treated with or without NMN-PEG. The RNA-seq data of HK-2 cells from different groups were clearly distinguished by PCA (n = 3 in each group). (B, D) Differentially expressed genes derived from HK-2 cells subjected to different treatments. The DEGs derived from HR-treated HK-2 cells *vs.* the control (B) and HR-stimulated cells treated with NMN-PEG *vs.* untreated cells (D) are shown as volcano plots. (C, E) KEGG analysis of the enriched pathways in HK-2 cells in response to different treatments. The top 20 enrichment pathways of HR-treated HK-2 cells *vs*. control (C) and HR-stimulated cells treated with NMN-PEG *vs*. untreated (E). (F) Heatmap analysis of the peroxisome-associated DEGs affected by NMN-PEGs in HR-treated HK-2 cells. On the basis of the RNA-seq data of HK-2 cells with different treatments, DEGs associated with peroxisome functions are displayed in a heatmap. (G) The expression of ACAA1 in HK-2 cells in different treatment groups. HR-treated HK-2 cells were treated with NMN-PEG, ACAA1 siRNA (siACAA1), or NMN-PEG/siACAA1, after which the mRNA level of ACAA1 was measured. The experiments were conducted in triplicate. (H) Protein expression levels of ACAA1 and PMP70 in HK-2 cells in different groups. HK-2 cells from different groups were lysed for western blotting using anti-ACAA1 and anti-PMP70 antibodies. (I) Immunofluorescence staining of peroxisomes in HK-2 cells after different treatments. HK-2 cells transfected with or without siACAA1 were subjected to HR stimulation, followed by immunofluorescence analysis of peroxisome (PMP70) in the control and HR-treated HK-2 cells (scale bar: 50 μm). The fluorescence intensity of PMP70 was quantified. (J) Lipid accumulation in HK-2 cells after different treatments. Oil red O staining (upper panel) and BODIPY 493/503 methyl bromide staining (lower panel) were used to detect lipid accumulation in HK-2 cells subjected to different treatments (scale bar: 10 μm). Both the oil red area and the number of lipid droplets were quantified. (K) Triglyceride content in HK-2 cells from different groups. (L, M) The role of NMN-PEG in alleviating HR-induced ROS production and lipid peroxidation in HK-2 cells was mediated by ACAA1. HK-2 cells were subjected to HR stimulation for 24 h after transfection with siACAA1 for 12 h and then subjected to NMN-PEG (10 μg/mL) treatment for 24 h before ROS and lipid peroxidation analysis. A DCFH-DA probe was used to detect ROS levels in HK-2 cells from different groups (L). BODIPY 581/591 C11 staining was performed to detect intracellular lipid peroxidation, which was quantified using the fluorescence intensity (M). (N) PEGylated NMN regulates peroxisomal function through ACAA1 to promote lipid metabolism. The data are presented as the means ± SDs. HR, hypoxia-reoxygenation.

**Figure 5:**
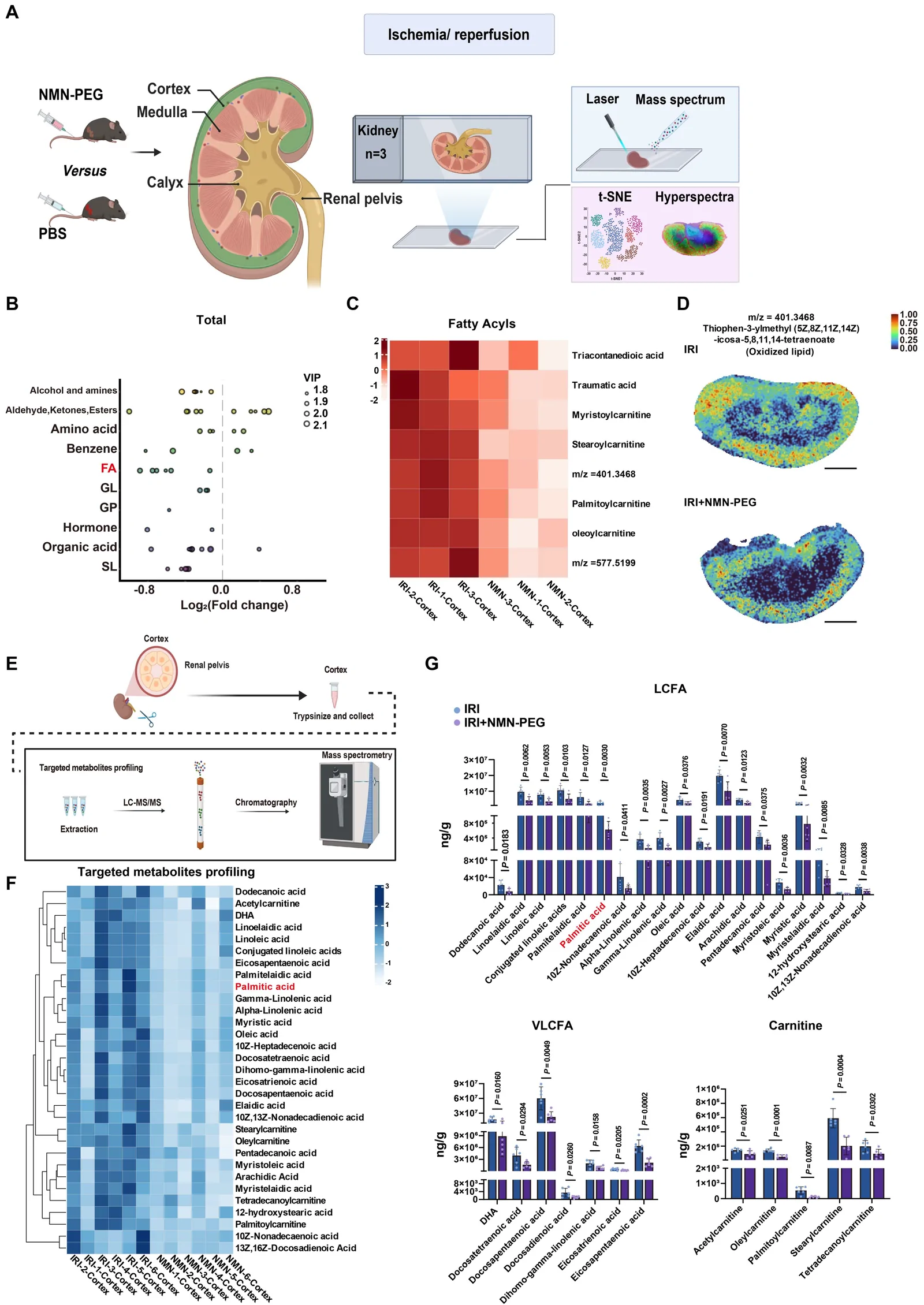
** MALDI‒MS-based spatial metabolism analysis and high-throughput targeted assays revealed the effect of PEGylated NMN on the fatty acid metabolomics of the renal cortex in AKI mouse models.** (A) Schematic of the MALDI‒MS-based spatial metabolism analysis of kidneys from AKI mice treated with or without NMN-PEG. After 48 h of NMN-PEG (20 mg/kg) or saline treatment, the kidneys of IRI-induced AKI mice were subjected to MALDI‒MS-based untargeted spatial metabolism analysis. (B) Differentially expressed metabolite classification of the kidney in response to NMN-PEG treatment in IRI mouse models. Bubble map of total differentially expressed metabolite classification in the kidneys of IRI-induced AKI mice. VIP: variable importance in projection. A VIP > 1 was considered to indicate a significant difference. (C) Heatmap of the differentially expressed metabolites of fatty acyls in the renal cortex. The differentially expressed metabolites of fatty acyls in the cortex of IRI mouse models treated with or without NMN-PEG were further analyzed using the heatmap, which revealed a significant inhibitory effect of NMN-PEG on the expression of fatty acyls in the renal cortex IRI mice. (D) The spatial expression of oxidized lipids in IRI mice treated with or without NMN-PEG is shown (scale bar: 50 μm). (E) Illustration of mouse kidney cortex extraction for targeted metabolomics. (F) Cluster heatmap analysis of metabolites whose expression significantly differed in the kidney cortex (n = 6). (G) Quantitative statistical analysis of major differentially expressed metabolites (n = 6). The data are presented as the means ± SDs.

**Figure 6:**
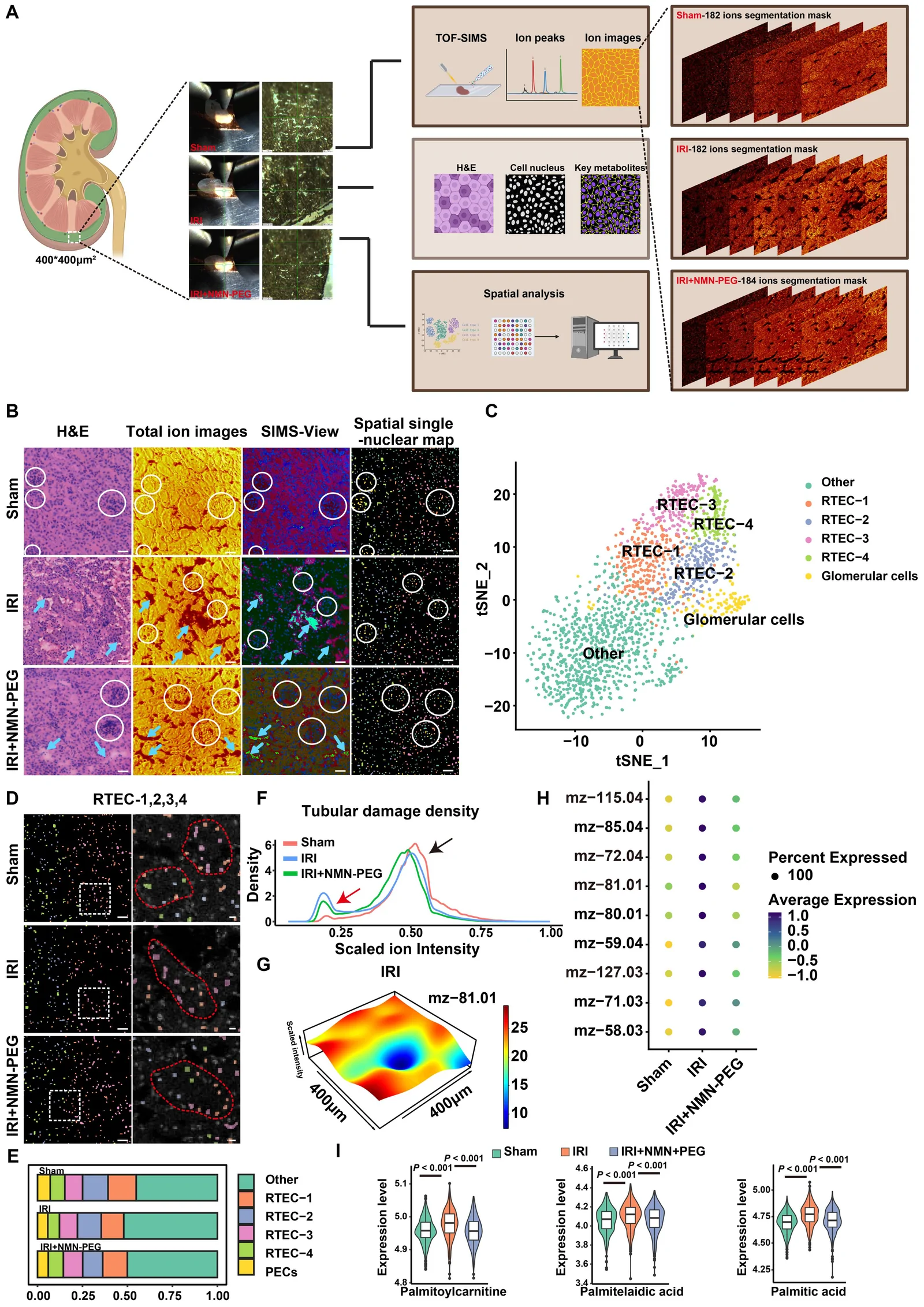
** Spatial single nuclear metabolomics (SEAM) analysis of the spatial expression pattern of metabolites in the kidneys of IRI mice upon NMN-PEG treatment.** (A) Schematic of the workflow of the SEAM analysis. In brief, freshly dissected kidney blocks were trimmed to a size of 50 mm × 20 mm to be cryopreserved in OCT and then cut into continuous tissue sections of 10-15 μm thickness before they were mounted onto positively charged slides for the TOF-SIMS assay. Ion signals and images and H&E staining were used to profile the spatial metabolite expression pattern. (B) Representative images of the spatial single-nuclear metabolite map. Representative H&E staining, total ion, and color-coded pixel visualization images from the SIMS view were matched within the same field of view to obtain a spatial single-nuclear metabolic map for *in situ* metabolic cell type visualization. Scale bar: 100 μm; white circles: different metabolic clusters of RTECs. (C) T-SNE plot of metabolic clusters of RTECs. Four clusters of renal tubular epithelial cells were identified by their differentially expressed metabolic profiles on the t-SNE plot. (D, E) Proportion of different metabolic clusters of RTECs in different mouse models. Representative images of one metabolic cluster of RTECs in the spatial single-nucleus map (D, left scale bar: 100 μm; right scale bar: 10 μm). The proportions of different metabolic clusters of cells in the kidneys of the sham and IRI mice treated with or without NMN-PEG (E). (F) Analysis of tubular damage density in mice with IRI upon NMN-PEG treatment. A scaled ion intensity curve was used to analyze renal tubular damage under different treatment conditions. (G) 3-dimensional surface visualization of the enriched metabolites within the damaged area. The metabolite of *m/z* 81.01 was revealed to be closely associated with renal tubular damage in IRI models. (H, I) Changes in ion species in the kidneys of mice from different groups. Changes in the expression of the differentially expressed ion species in the kidneys of mice subjected to different treatments are shown in a dot plot (H). The levels of palmitoylcarnitine, palmitelaidic acid, and palmitic acid were analyzed using violin plots (I). The data are presented as the means ± SDs.

**Figure 7:**
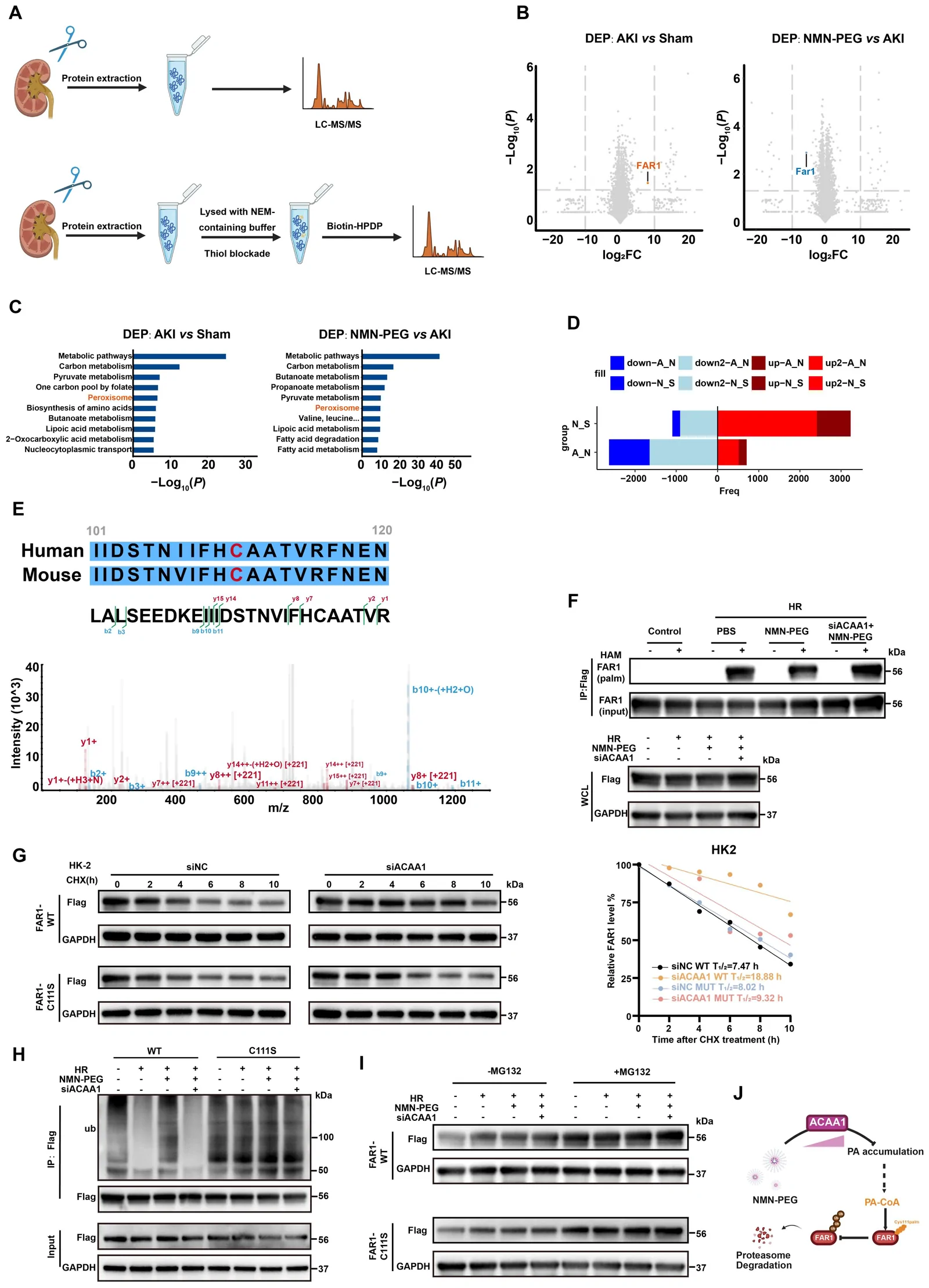
** Quantitative proteomics revealed that NMN-PEG-mediated ACAA1 upregulation led to impaired FAR1 palmitoylation.** (A) Schematic of the workflow of the quantitative proteomics analysis. In brief, mouse kidney tissues were pulverized in liquid nitrogen and homogenized in lysis buffer containing 4% SDS, 100 mM Tris-HCl (pH 8.0), 5 mM EDTA, and a protease inhibitor cocktail. The lysates were sonicated on ice for 2 min, followed by centrifugation at 4 °C to collect the supernatants. Following protein quantification, equal amounts of protein from each sample were subjected to SDS‒PAGE electrophoresis, and the samples were then processed for palmitoylation analysis and LC‒MS/MS analysis. (B) Volcano plot analysis of differential loci between AKI *vs.* sham and AKI treated with NMN-PEG *vs.* AKI. The *x*-axis shows the log2-fold change at the protein level, while the* y*-axis represents the significance as -log10 of the Benjamini-Hochberg (BH)-corrected *p* values derived from a two-sided Welch *t* test. (C) KEGG pathway enrichment analysis of differential loci in the sham *vs.* AKI and NMN-PEG-treated *vs.* AKI groups. (D) Distribution plot of differential loci in the sham *vs.* AKI and NMN-PEG-treated *vs.* AKI groups. Differentially significant loci were identified using Student's t test (*p* value < 0.05) combined with fold change (FC) thresholds (FC > 2.0 or < 0.5). (E) Protein sequence alignment of human and mouse Far1 using EMBOSS Matcher and peptide mass fingerprinting of palmitoylated site of Far1 by LC‒MS/MS data. (F) ABE and western blot analyses of FAR1 palmitoylation induced by HR in HK-2 cells. Control HK2 cells and cells treated by NMN-PEG or siACAA1 plus NMN-PEG upon HR were subjected to ABE analysis. (G) Immunoblot (left) and linear regression (right) analyses of the effects of siACAA1 on FAR1 protein degradation in HK-2 cells transfected with FLAG-FAR1-WT or FLAG-FAR1-C111S mutant plasmids for 48 h. The transfected cells were then treated with cycloheximide (CHX, 200 mg/mL) for indicated times before subjected to Western blot analysis. (H) Ubiquitin analysis of FLAG-FAR1-WT- and FLAG-FAR1-C111S- transfected HK-2 cells under different treatments. The WT and C111S cells were subjected to NMN-PEG or NMN-PEG plus siACAA1 transfection and then immunoprecipitated with anti-Flag antibody, followed by ubiquitin analysis. (I) Immunoblot analysis of FAR1 protein levels in HK-2 cells under different treatments. HK2 cells were transfected with FLAG-FAR1-WT or FLAG-FAR1-C111S for 48 h, and then treated with MG132 (20 μM) for 6 hours before harvested for protein analysis. (J) The diagram of the molecular mechanism underlying NMN-PEG-mitigated ferroptosis in AKI. Briefly, HR-induced FAR1 palmitoylation at Cys111 was suppressed by NMN-PEG through ACAA1-mediated conversion of palmitic acid to palmitoyl-CoA (P-CoA), thus promoting FAR1 ubiquitination and proteasomal degradation.

**Figure 8:**
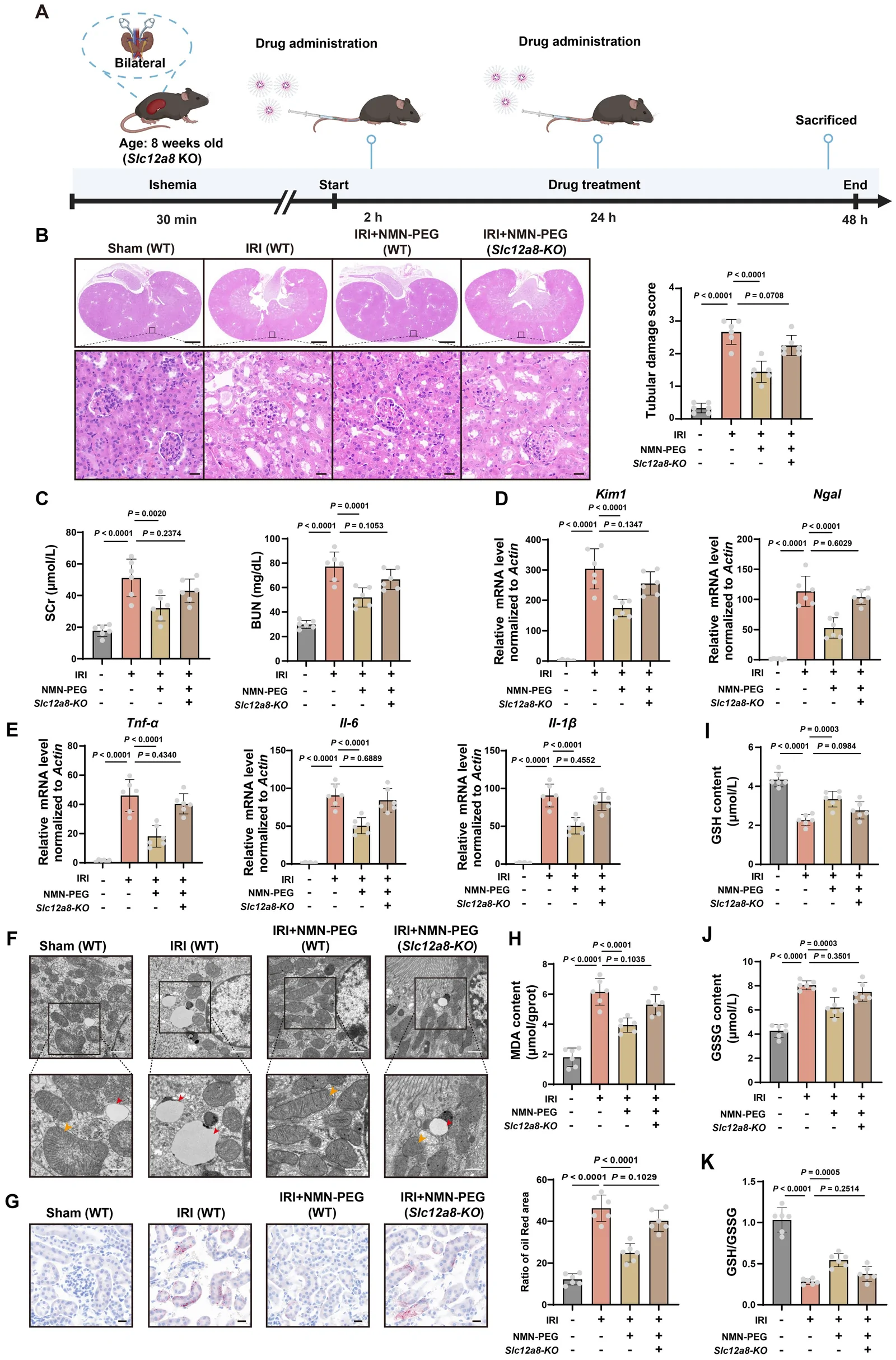
** The cellular transport of NMN-PEG relied on SLC12A8 protein.** (A) The schematic of the construction of IRI-induced AKI mouse model and NMN administration in the WT and *Slc12a8*-knockout (KO) mice. (B-E) The analyses of the effect of NMN-PEG on AKI in the WT and *Slc12a8-*KO mouse models. Representative H&E images of the kidney (n = 6; upper scale bar: 1000 μm; lower scale bar: 20 μm) were shown and quantified by the scoring of renal tubular damage (B). The serum levels of blood urea nitrogen (BUN) and serum creatinine (SCr) in the mice in the different groups were measured (C, n = 6). The relatively mRNA levels of AKI biomarkers (D, *Kim1* and* Ngal*) and inflammatory cytokines (E, *Tnf-α*,* Ii-6*, and *Il-1β*) in the kidneys of the WT and *Slc12a8-*KO mice treated with or without NMN-PEG in response to IRI treatment were examined (n = 6). (F-K) Analyses of the effect of NMN-PEG on AKI-induced ferroptosis in WT and *Slc12a8-*KO mice models. Representative TEM images of kidney mitochondria from different groups of mice (F, upper scale bar: 1.0 μm; lower scale bar: 0.5 μm). Mitochondria are indicated by yellow arrows. Lipid droplet is indicated by red arrows. Representative Oil Red O-staining images of kidney in different groups of mice (G, scale bar: 20 μm). The content of MDA in the kidney tissues of the mice was analyzed (H). Renal expression of GSH and GSSG and the ratio of GSH/GSSG in mice from different groups (I-K). (n = 6). The data are presented as the means ± SDs.

## Abbreviations

AKI: Acute kidney injury
LPO: Lipid peroxidation
PTCs: Proximal tubule cells
LINCS: Library of Integrated Network-based Cellular Signatures
MTDS: Mouse Tissue-specific Drug Signatures
NMN: Nicotinamide mononucleotide
PEG: Polyethylene glycol
ACAA1: Acetyl-CoA acyltransferase 1
PPAR: Peroxisome proliferator-activated receptor
LCFAs: Long-chain fatty acids
VLCFAs: Very-long-chain fatty acids
MALDI-MS: Matrix-assisted laser desorption/ionization-mass spectrometry
SIMS: Secondary ion mass spectrometry
SEAM: Single nuclear metabolomics
FAR1: Fatty acyl-CoA reductase 1
LPS: Lipopolysaccharide
SCr: Serum creatinine
BUN: Blood urea nitrogen
ABE: Acyl-biotin exchange
DEGs: Differentially expressed genes
GO: Gene ontology
KEGG: Kyoto Encyclopedia of Genes and Genomes
NMN-PEG: PEGylated NMN nanoparticles
IRI: Ischemia reperfusion injury
HR: Hypoxia-reoxygenation
GSH: Glutathione
GSSG: Glutathione disulfide
MDA: Malondialdehyde
KIM-1: Kidney injury molecule 1
NGAL: Neutrophil gelatinase-associated lipocalin
RTECs: Renal tubular epithelial cells
FAO: Fatty acid oxidation
