# Supplementary material for: PEGylated nicotinamide mononucleotide targeted ferroptosis in acute kidney injury by suppressing FAR1 palmitoylation in an ACAA1-dependent manner
Source: Int J Biol Sci. 2026 Oct 2;22(15):8614–35. doi: 10.7150/ijbs.132234 (PMC13685766; doi:10.7150/ijbs.132234)
Supplement: Supplementary file 1 [file ijbsv22p8614s1.pdf]

PEGylated nicotinamide mononucleotide targeted ferroptosis in acute kidney injury by suppressing FAR1 palmitoylation in an ACAA1-dependent manner

Authors: Yang Yue[1 #], Changdong Zhou[1,4#], Qingyu Li[1 #], Xiaochang Zhang[1#], Hongjun Lin[1], Ming Niu[5], Aihong Zhang[6], Shuiqin Gong[6], Lijing Yang[7], Yuke Cui[4], Hong Chen[1], Xiaodan Cheng[1], Jiangbo Li[1], Qi He[1], Chenhui Wang[1], Jian Li[1], Wen Yang[1], Jian Bai[1], Jingqi Shi[1], Yanze Li[8], Xing Qin[8], Xiangmei Chen[2,3*], Liang Guo[1*], Shengqi Wang[1, 3*]

1. Academy of Military Medical Sciences, Beijing 100850, China
2. Department of Nephrology, First Medical Center of Chinese PLA General Hospital, Beijing 100853, China
3. National Key Laboratoty of Kidney Diseases, National Clinical Research Center of Kidney Diseases, Beijing 100853, China
4. Pingyuan Laboratory, Henan 453000, China
5. Department of Hematology, the Fifth Medical Center of Chinese PLA General Hospital, Beijing 100071, China
6. Department of Nephrology, Chongqing Key Laboratory of Prevention and Treatment of Kidney Disease, Chongqing Clinical Research Center of Kidney and Urology Diseases, Xinqiao Hospital, Army Medical University (Third Military Medical University), Chongqing 400037, China
7. Medical Service Training Center, Chinese PLA General Hospital, Beijing 100853, China
8. CapitalBio Technology Corporation, Beijing 101111, China

# Contributed equally: Yue Yang, Changdong Zhou, Qingyu Li, Xiaochang Zhang

*Correspondence: Liang Guo, Xiangmei Chen, Shengqi Wang

corresponding authors e-mail address: gllsunmo369@sina.com, xmchen301@126.com, sqwang@bmi.ac.cn,

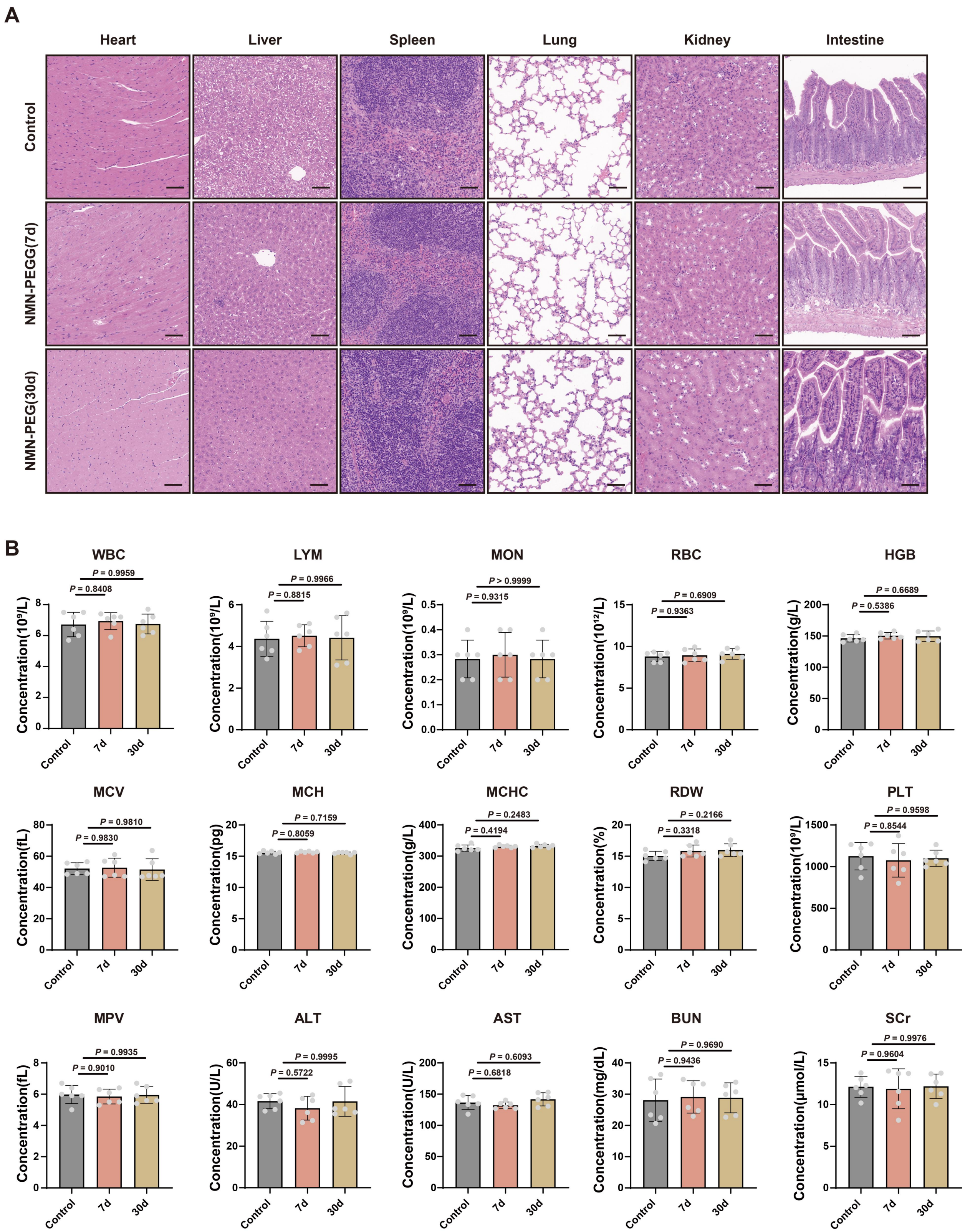

Fig S1. *In vivo* toxicity assessment of PEGylated-NMN. (A) H&E staining of major organs in mice on days 7 and 30 after PEGylated-NMN (20mg/kg) treatment for two consecutive days. Scale bar

= 50 μm. (B) Routine blood examination, liver and renal function were measured from mice in (A) (n = 6). ns. no significant. WBC, white blood cell; LYM, lymphocyte; MON, monocyte count; RBC, red blood cell; HGB, hemoglobin; MCV, mean corpuscular volume; MCH, mean corpuscular hemoglobin; MCHC, MCH concentration; RDW, red cell distribution width; PLT, platelet; MPV, mean platelet volume; ALT, alanine transaminase; AST, aspartate transaminase; BUN, blood urea nitrogen; SCR, serum creatinine.

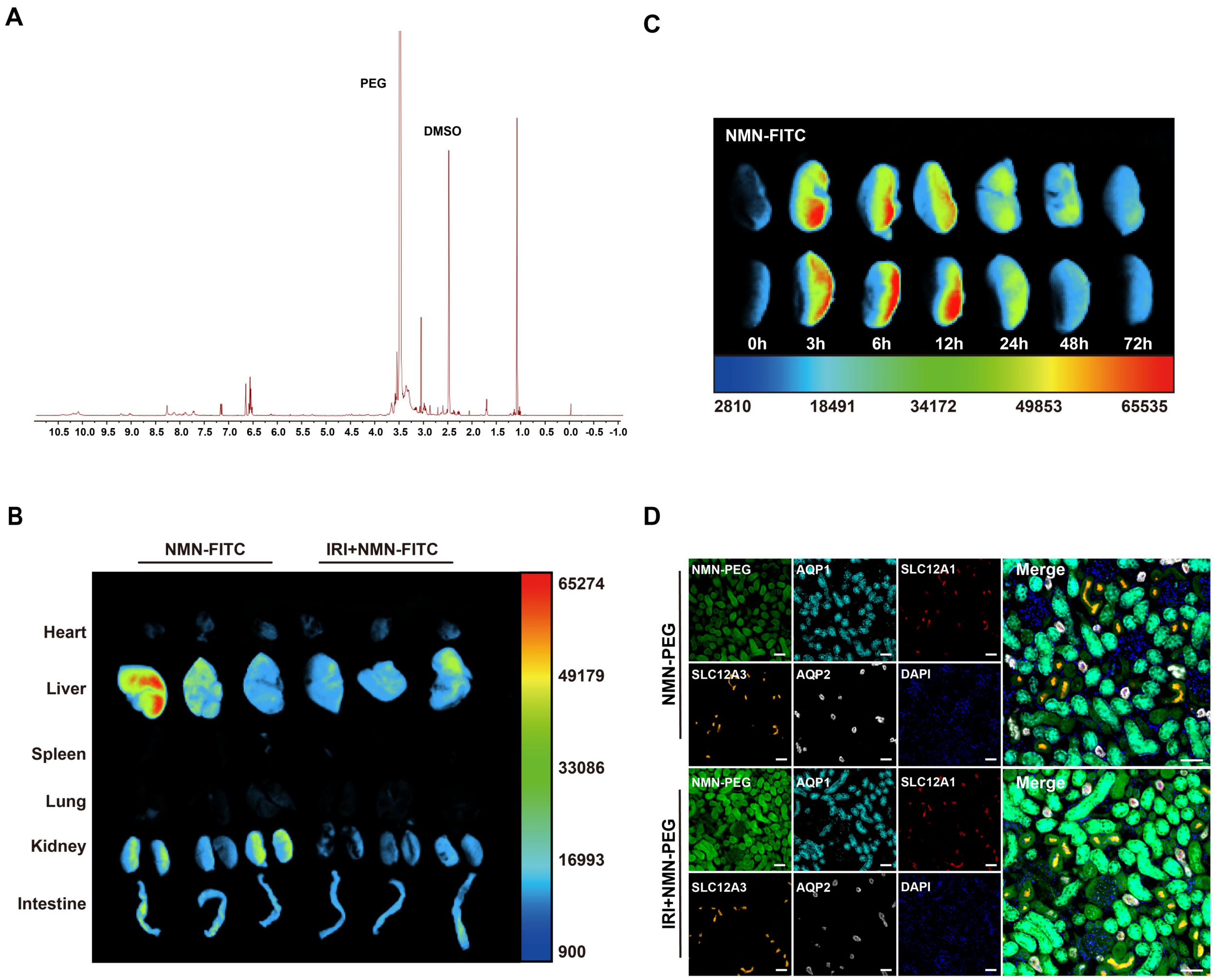

Fig S2. Preferential uptake of PEGylated NMN in the cortex of the kidney in AKI mouse models. (A) NMR spectrum of NMN-PEG. (B) Imaging of major organs in mice after injection of FITC-labeled NMN. (C) Representative fluorescence images of the kidneys of mice at different time

points (3, 6, 12, 24, 48, and 72 h) after the injection of FITC-labeled NMN. (D) Representative fluorescent images of PEGylated NMN, AQP1, AQP2, SLC12A1 and SLC12A3 in the kidneys of NMN-PEG-treated control and AKI mice are also displayed. Scale bar: 20 μm.

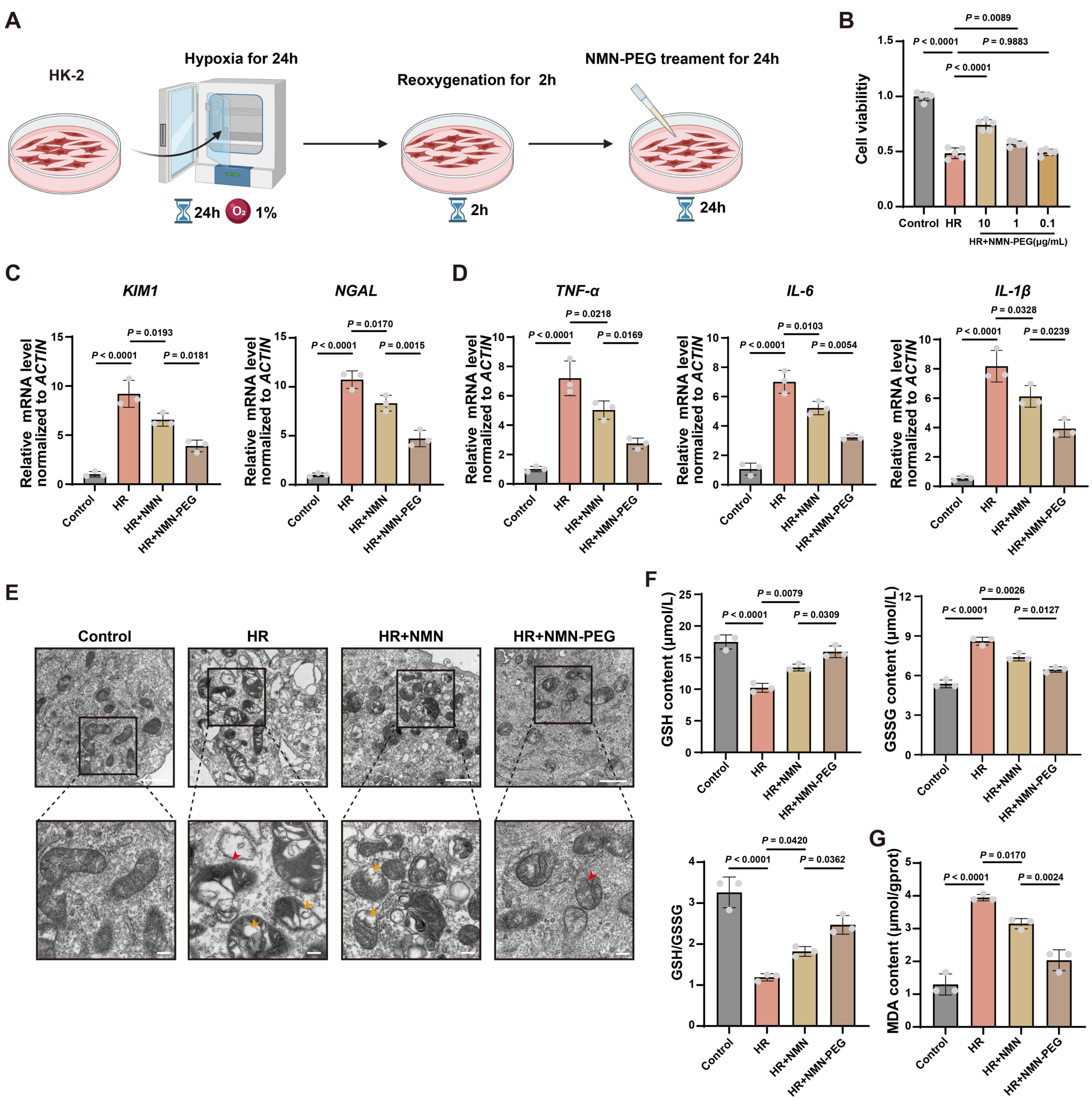

Fig S3. PEGylated-NMN alleviated HR induced ferroptosis *in vitro*. (A) Diagram of HR and PEGylated-NMN treatment in HK-2. (B) Effects of different concentrations of PEGylated-NMN

on the viability of HK-2 after HR (n = 6). (C, D) Relative mRNA expression of *KIM1*, *NGAL*, *TNF-α*, *IL-6* and *IL-1β* in HK-2 (n = 3). (E) Representative TEM images of HK-2 cells (left panel scale bar = 1.0 μm; right panel scale bar = 0.5μm). Mitochondrial outer membrane rupture or mitochondrial crest injury are indicated by yellow arrows. Mitochondrial shrinkage is indicated by red arrows. (F) GSH, GSSG and GSH/GSSG levels in HK-2 (n = 3). (G) MDA levels in HK-2 (n = 3). The data are presented as mean ± SDs. HR, hypoxia-reoxygenation.

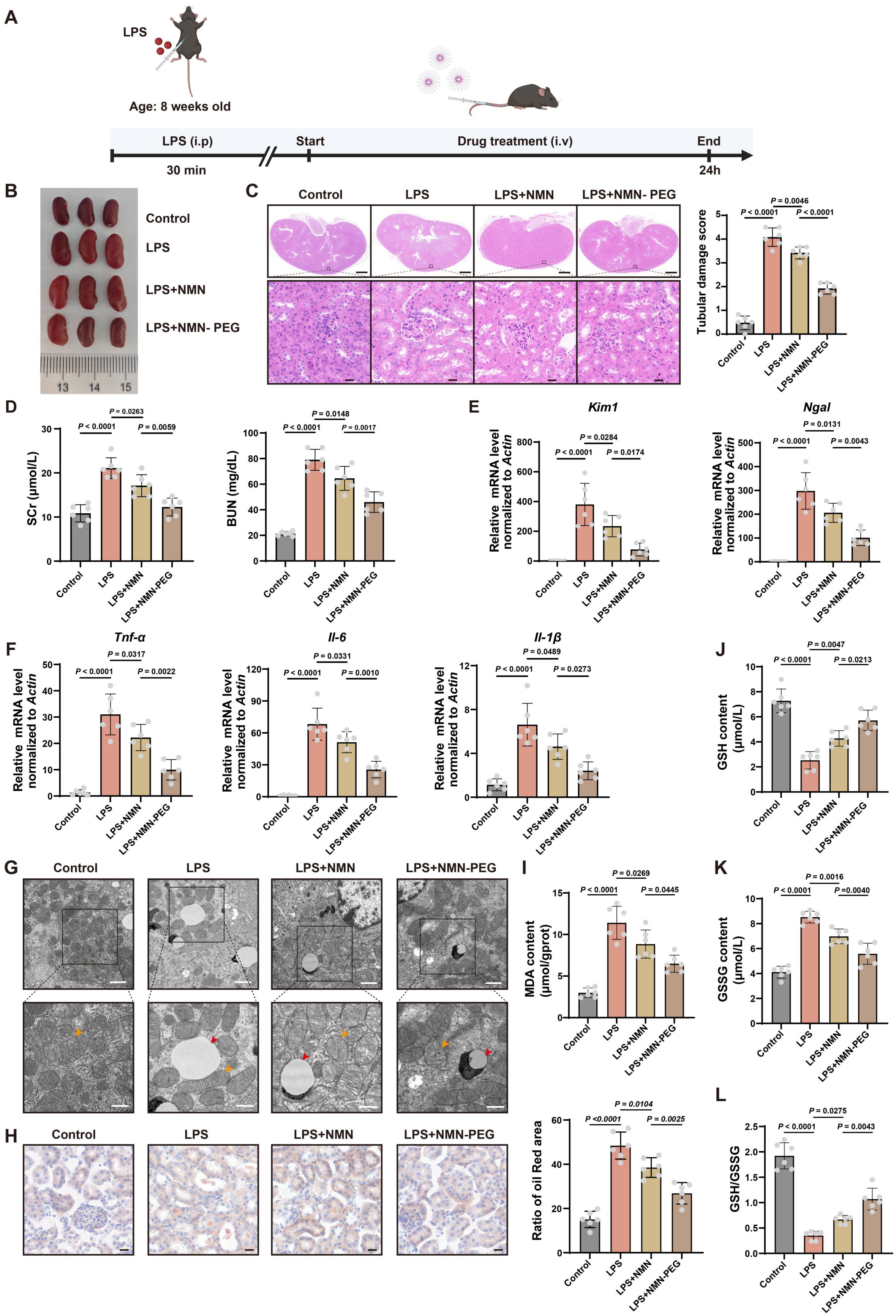
A
LPS
Age: 8 weeks old
LPS (i.p)
Start
Drug treatment (i.v)
End
30 min
24h
B
Control
LPS
LPS+NMN
LPS+NMN- PEG
C
Control
LPS
LPS+NMN
LPS+NMN- PEG
Tubular damage score
D
SCr (μmol/L)
BUN (mg/dL)
E
Kim1
Ngal
Relative mRNA level normalized to Actin
F
Tnf-α
Il-6
Il-1β
G
H
I
MDA content (μmol/gprot)
Ratio of oil Red area
J
GSH content (μmol/L)
K
GSSG content (μmol/L)
L
GSH/GSSG
Control
LPS
LPS+NMN
LPS+NMN-PEG

55

Fig S4. PEGylated-NMN alleviated LPS-induced pathological changes and ferroptosis *in vivo.* (A) Diagram of LPS and PEGylated-NMN treatment *in vivo.* (B) The macroscopic and H&E images of the kidney. (C) Representative images of H&E staining (up panel scale bar = 1000 μm; down panel scale bar = 20 μm). Tubular damage was scored according to H&E staining (n = 6). (D) The serum levels of SCr and BUN from mice (n = 6). (E, F) Relative mRNA expression of *Kim1*, *Ngal*, *Tnf-α*, *Ii-6*, *Il-1β* in kidney tissues (n = 6). (G) Representative TEM images of kidney mitochondria from different groups of mice (upper scale bar: 1.0 μm; lower scale bar: 0.5 μm). Mitochondria are indicated by yellow arrows. Lipid droplets are indicated by red arrows. (H) Representative Oil Red O-staining images of kidney in different groups of mice (scale bar: 20 μm). (I) The content of MDA in the kidney tissues of the mice was analyzed (n = 6). (J–L) Renal expression of GSH and GSSG and the ratio of GSH/GSSG in mice from different groups (n = 6). The data are presented as mean ± SDs.

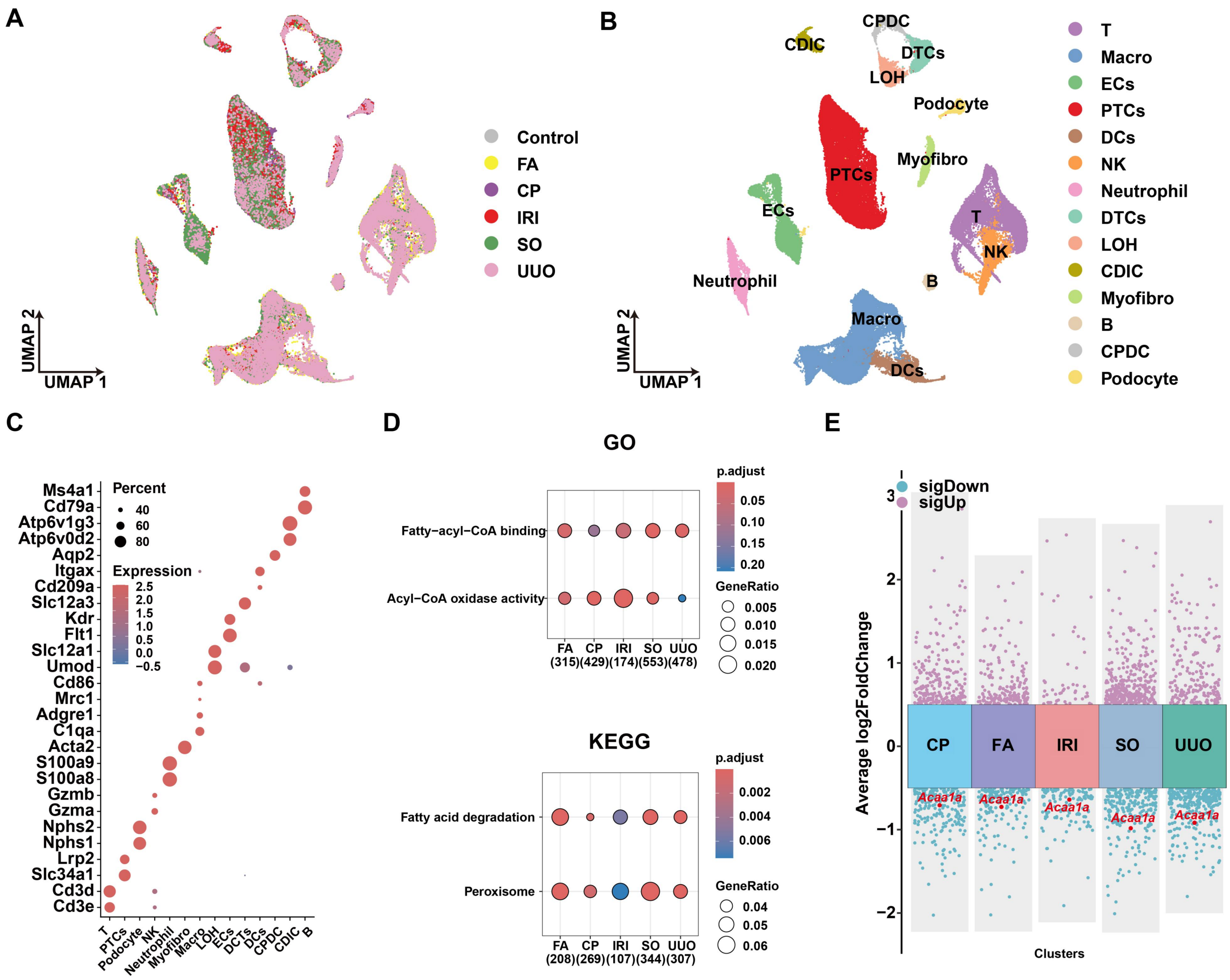

Fig S5. scRNA-seq identifies ACAA1 downregulation as a conserved metabolic node in AKI pathogenesis. (A) UMAP visualization of scRNA-seq data of the kidney derived from multiple AKI mice models. The previously published scRNA-seq data of the kidneys from five common AKI etiologies (CP: cisplatin, IRI: ischemia-reperfusion injury, UUO: unilateral ureteral obstruction, FA: folic acid, SO: sodium oxalate) were analyzed and visualized by UMAP. (B, C) Cell clustering of the scRNA-seq data by signature genes. The UMAP (B) and dot plot (C) analysis revealed 14 clusters of different cell types according to the signature genes by the scRNA-seq data. (D) The GO and KEGG analysis of the enriched pathways in different AKI mice models. Fatty acid metabolism and peroxisome function were enriched in all the AKI models. (E) Box plots of

renal DEGs in the five AKI models. ACAA1 was revealed to be significantly downregulated in all the AKI models.

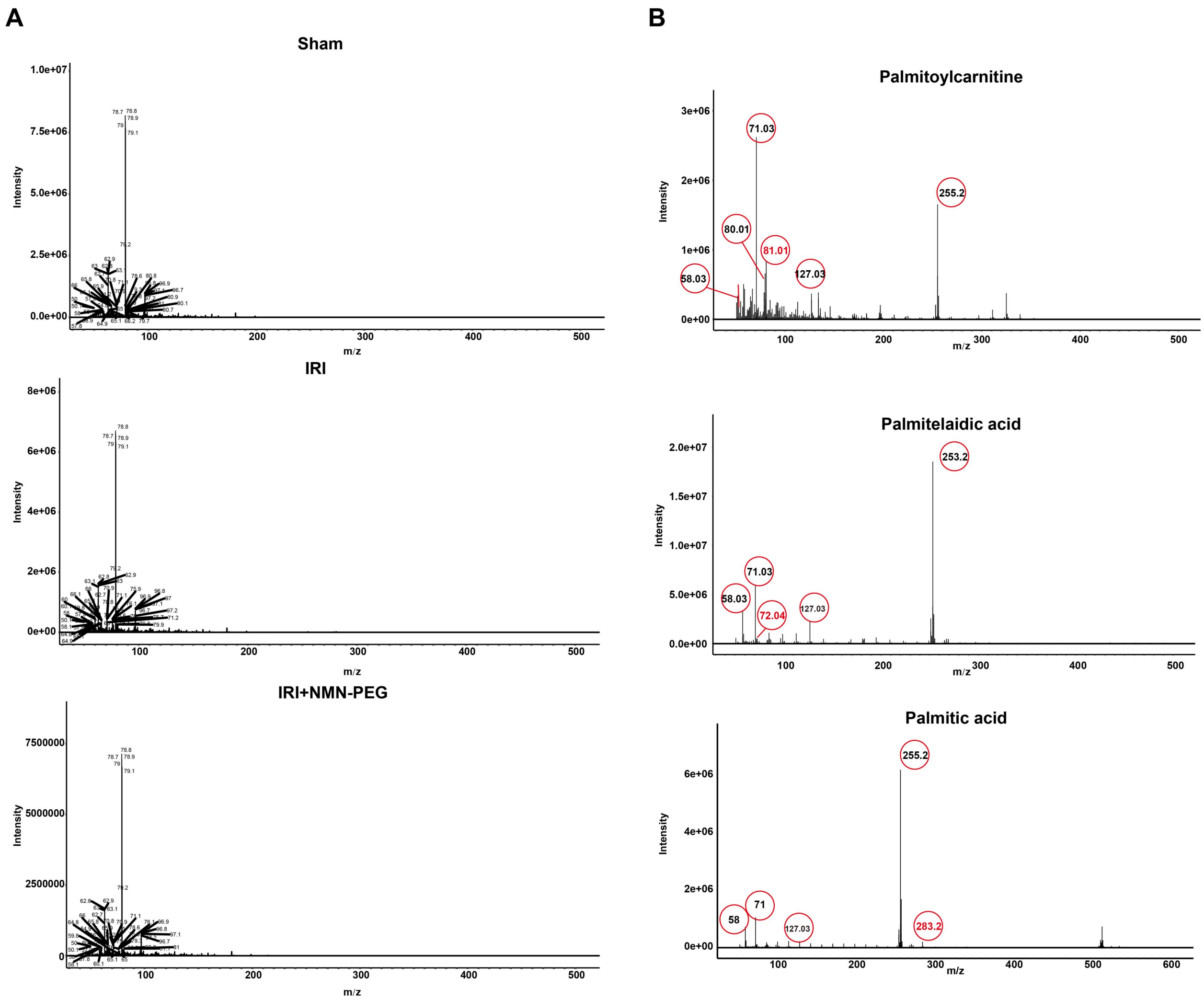

Fig S6. Intensity diagram of different ion species. (A) Intensity plot shows that ion species with zonation pattern stand out among all peaks. (B) Mass spectrum peaks of palmitoylcarnitine, palmitelaidic acid and myristic acid standard.

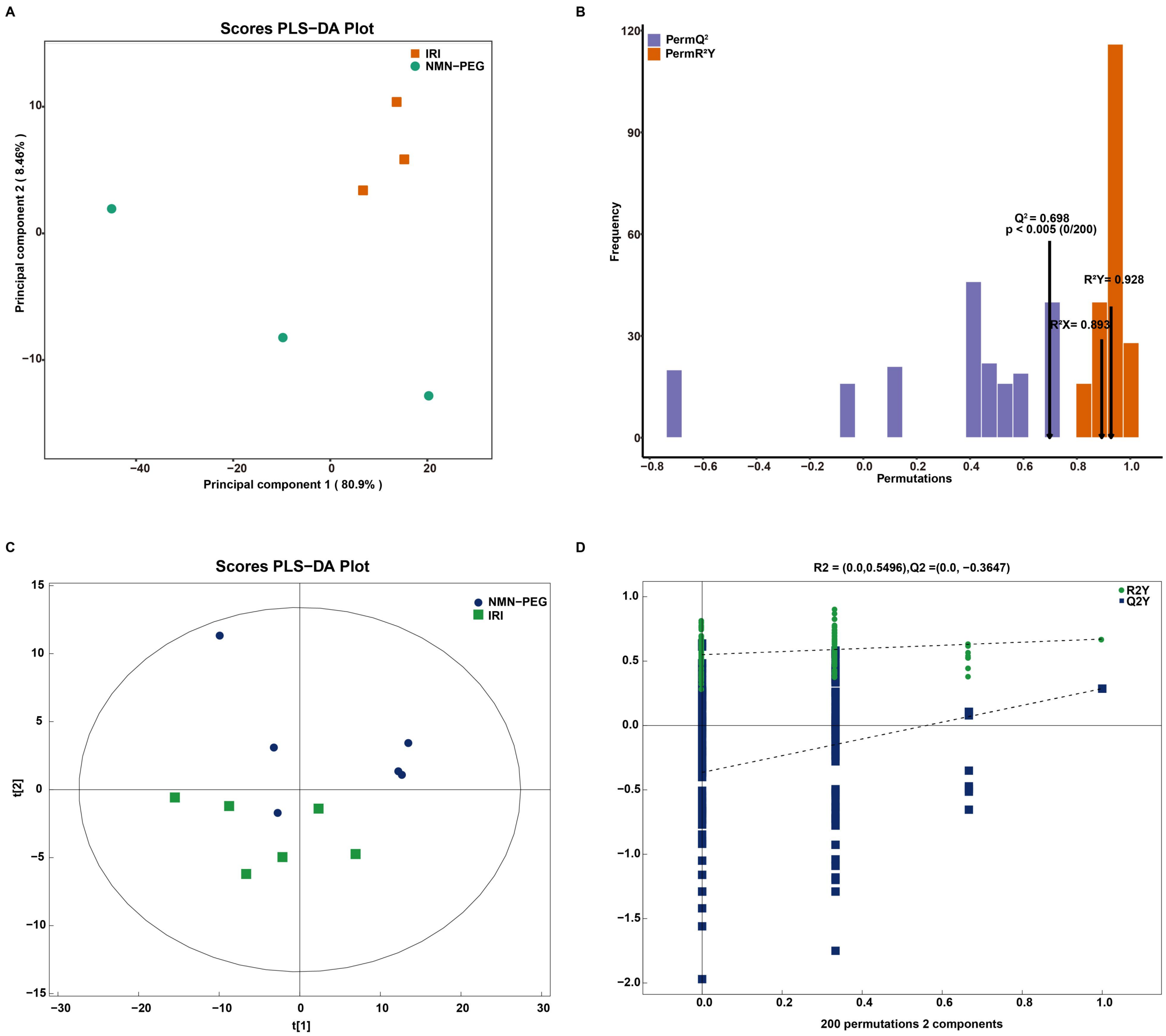

Fig S7. Principal component analysis of metabolomics. (A) The PLS-DA analysis of MALDI-MSI-based spatial metabolomics. (B) OPLS-DA permutation diagram of MALDI-MSI-based spatial metabolomics. (C) The PLS-DA analysis of targeted metabolomics of renal cortex. (D) Permutation diagram of targeted metabolomics of renal cortex.

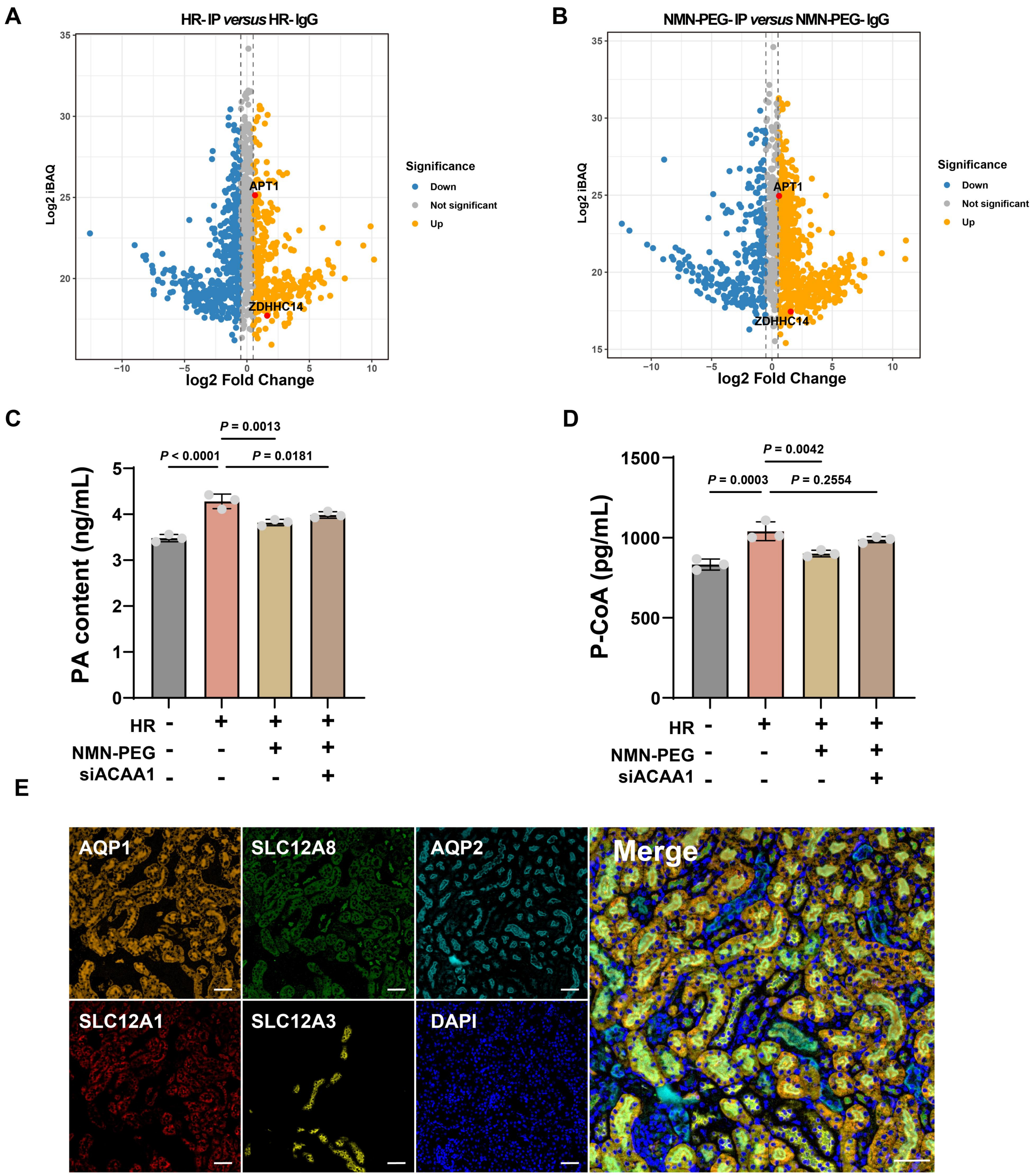

Fig S8. ACAA1 suppresses FAR1 palmitoylation by depleting its metabolic substrate, Palmitoyl-CoA. (A&B) Volcano plots from IP-MS analysis assessing the abundance of candidate palmitoylation enzymes. No significant changes in APT1 and ZDHHC14 levels were observed in HR group, or in NMN-PEG group. (C) Quantification of intracellular palmitic acid levels (n = 3).

(D) Quantification of intracellular Palmitoyl-CoA (P-CoA) levels (n = 3). (E) Representative multi-fluorescent images of SLC12A8, AQP1, AQP2, SLC12A1 and SLC12A3 in the kidneys of IRI-treated AKI mice. Scale bar: 20 μm. The data are presented as mean ± SDs.

Table S1. The list of mouse primer sequences

Mouse Gene	Sequence
F-Tnf-α	GATCGGTCCCCAAAGGGATG
R-Tnf-α	CACTTGGTGGTTTGTGAGTGT
F-Il-6	TTCCTCTGGTCTTCTGGAGT
R-Il-6	TCTGTGACTCCAGCTTATCTCTTG
F-Il-1β	ATGCCACCTTTTGACAGTGATG
R-Il-1β	TGATGTGCTGCTGCGAGATT
F-Ngal	GGAACGTTTCACCCGCTTTG
R-Ngal	CCACACTCACCACCCATTCA
F-Kim1	GTCAGCATCTCTAAGCGTGG
R-Kim1	TCTTCAGCTCGGGAATGCAC
F-β-actin	CACCCTGTGCTGCTCACC
R-β-actin	GAAGGTCTCAAACATGATCTGGGT

Table S2. The list of human primer sequences

Human Gene	Sequence
F-TNF-α	GCCCATGTTGTAGCAAACCCT
R-TNF-α	GGACCTGGGAGTAGATGAGGT
F-IL-6	AACTCCTTCTCCACAAGCGCC
R-IL-6	GAAGAGGTGAGTGGCTGTCTG
F-IL-1β	CCCTAAACAGATGAAGTGCTCCT
R-IL-1β	CTTGCTGTAGTGGTGGTCGG
F-NGAL	TCACCCTCTACGGGAGAACC
R-NGAL	GGTCGATTGGGACAGGGAAG
F-KIM1	GCCACTTCACCATCTTCACC
R-KIM1	ACGGTGTCATTCCCATCTGTT
F-ACAA1	GGCATGGCCTGTGGGATAA
R-ACAA1	TCTCAGCTTGGAAACAGCCC
F-β-ACTIN	AGGCACCAGGGCGTGA
R-β-ACTIN	CGATGGGGTACTTCAGGGTG
